# Right Ventricle Has Normal Myofilament Function But Shows Perturbations in the Expression of Extracellular Matrix Genes in Patients With Tetralogy of Fallot Undergoing Pulmonary Valve Replacement

**DOI:** 10.1161/JAHA.119.015342

**Published:** 2020-08-01

**Authors:** Daniel Brayson, So‐Jin Holohan, Sonya C. Bardswell, Matthew Arno, Han Lu, Hanna K. Jensen, Phan Kiet Tran, Javier Barallobre‐Barreiro, Manuel Mayr, Cristobal G. dos Remedios, Victor T. Tsang, Alessandra Frigiola, Jonathan C. Kentish

**Affiliations:** ^1^ School of Cardiovascular Medicine and Sciences King's College London BHF Centre for Research Excellence London United Kingdom; ^2^ Genomics Centre Faculty of Life Sciences and Medicine King’s College London London United Kingdom; ^3^ Great Ormond Street Hospital London United Kingdom; ^4^ Department of Anatomy Bosch Institute University of Sydney New South Wales Australia; ^5^ Guys and St Thomas’ NHS Foundation Trust St Thomas’ Hospital London United Kingdom; ^6^ School of Biomedical Engineering and Imaging Sciences Kings College London United Kingdom

**Keywords:** extracellular matrix, myofibril, pulmonary valve replacement, small leucine rich proteoglycan, tetralogy of Fallot, Cardiovascular Surgery

## Abstract

**Background:**

Patients with repair of tetralogy of Fallot (rToF) who are approaching adulthood often exhibit pulmonary valve regurgitation, leading to right ventricle (RV) dilatation and dysfunction. The regurgitation can be corrected by pulmonary valve replacement (PVR), but the optimal surgical timing remains under debate, mainly because of the poorly understood nature of RV remodeling in patients with rToF. The goal of this study was to probe for pathologic molecular, cellular, and tissue changes in the myocardium of patients with rToF at the time of PVR.

**Methods and Results:**

We measured contractile function of permeabilized myocytes, collagen content of tissue samples, and the expression of mRNA and selected proteins in RV tissue samples from patients with rToF undergoing PVR for severe pulmonary valve regurgitation. The data were compared with nondiseased RV tissue from unused donor hearts. Contractile performance and passive stiffness of the myofilaments in permeabilized myocytes were similar in rToF‐PVR and RV donor samples, as was collagen content and cross‐linking. The patients with rToF undergoing PVR had enhanced mRNA expression of genes associated with connective tissue diseases and tissue remodeling, including the small leucine‐rich proteoglycans ASPN (asporin), LUM (lumican), and OGN (osteoglycin), although their protein levels were not significantly increased.

**Conclusions:**

RV myofilaments from patients with rToF undergoing PVR showed no functional impairment, but the changes in extracellular matrix gene expression may indicate the early stages of remodeling. Our study found no evidence of major damage at the cellular and tissue levels in the RV of patients with rToF who underwent PVR according to current clinical criteria.

**Nonstandard Abbreviations and Acronyms**
CMR: cardiac magnetic resonance
ECM: extracellular matrix
EF: ejection fraction
GO: gene ontology
LV: left ventricle
NYHA: New York Heart Association
PVR: pulmonary valve replacement
PCR: polymerase chain reaction
rToF: repair of tetralogy of Fallot
RV: right ventricle
SLRP: small leucine rich proteoglycan

**Clinical Perspective**

**What Is New?**

- We found that contractile function of the myofilaments, taken at the time of pulmonary valve replacement (PVR), in the right ventricular myocardium of patients with repaired tetralogy of Fallot was similar to that of age‐matched donor controls.
- Similarly, the extracellular collagen matrix was similar to that in donor control tissue, but there were subtle changes in gene expression, particularly in small leucine‐rich proteoglycans, although these changes were not reproduced at the protein level.

**What Are the Clinical Implications?**

- The optimal timing for PVR in patients with repaired tetralogy of Fallot is controversial.
- It has been suggested that right ventricular dilation over the years preceding PVR could lead to pathologic changes at the tissue and cellular levels that would limit long‐term recovery after PVR.
- We found of no evidence of myocyte or extracellular matrix remodeling, which suggests that patients are not exposed to detrimental or irreversible tissue remodeling with current PVR timing guidelines; however, the subtle changes in gene expression may indicate an impending program of myocardial remodeling.

Tetralogy of Fallot (ToF) is one of the most common congenital heart diseases, accounting for almost 10% of all cardiac malformations.^1^ Without surgical intervention, only 10% to 15% of patients survive beyond the age of 20 years. The modern surgical approach for these patients is for a single‐stage repair of ToF (rToF) performed during the first few months of life, which has led to excellent long‐term survival (>98% survival at 20 years after repair).^2^ Nevertheless, residual hemodynamic lesions remain and need to be addressed to avoid long‐term detrimental effects. The most common defect is pulmonary regurgitation, which is known to have a deleterious effect on global cardiac function, leading to progressive right ventricle (RV) dilatation and dysfunction, tricuspid valve regurgitation, exercise intolerance, arrhythmias, and sudden death.^3^, ^4^, ^5^, ^6^ Consequently, pulmonary valve replacement (PVR) is often advocated for the lifetime management of these patients. PVR has been shown in numerous studies to induce beneficial remodeling, not only of the RV but also the left ventricle (LV),^7^, ^8^, ^9^ coupled with improvement in patients' symptoms and exercise capacity.^7^ However, the optimal timing for PVR is controversial. To date, all commercially available pulmonary valves, whether surgically or percutaneously implanted, have limited durability, exposing patients to the need for multiple procedures during their lifetime. Although the number of repeat procedures could be reduced by delaying PVR, any delay could also theoretically lead to irreversible changes in RV structure and function, potentially resulting in poor long‐term recovery in some patients after PVR. Therefore, the timing of PVR is critical.

In previous studies, we and others demonstrated post‐PVR positive remodeling, characterized by reduction of RV volumes, increase in LV filling, and improvement of biventricular systolic function across a range of ages, but the greatest improvement of LV dynamics and exercise capacity was observed in younger patients (aged <18 years). On preoperative cardiac magnetic resonance (CMR) imaging and echocardiographic assessment, the only difference we observed between younger and older patients was a higher pulmonary valve regurgitant fraction in the younger group, which we interpreted to be the result of a more compliant, less “diseased” RV in younger patients.^7^ However, this suggestion highlighted a major deficiency in knowledge in this field: very little is known about the nature and extent of the pathologic tissue changes that take place in the dilated RV as a result of severe pulmonary valve regurgitation. In other words, by the time PVR is performed, how severe is the cellular and tissue damage in the RV?

Chronic volume overload of the LV (eg, due to myocardial infarction or aortic regurgitation) is associated with changes in the passive stiffness and contractile function of myocytes and in the microarchitecture of the extracellular matrix (ECM) that may either contribute to, or compensate for, mechanical dysfunction.^10^, ^11^, ^12^ However, it is not known if similar pathologies exist in the volume‐overloaded RV myocardium ofpatients with rToF at the time of PVR. In this study, we sought to assess the contractile properties and passive stiffness of the cardiac myofilaments and the composition of the ECM in these patients undergoing PVR to better understand the functional properties at the cellular level of the dilated RV with significant pulmonary regurgitation. In the long term, improved understanding of the nature and extent of maladaptive RV remodeling may help inform therapeutic strategies and aid clinical decisions regarding the time at which PVR should be performed in individual patients with rToF.

## Methods

### Data Availability Statement

The data that support the findings of this study are available from the corresponding authors on reasonable request. The microarray data are publicly accessible at the National Center for Biotechnology Information (NCBI) Gene Expression Omnibus (GEO) database (https://www.ncbi.nlm.nih.gov/geo/) under accession number GSE141955.

### Ethical Approval

The project was approved by the UK National Research Ethics Committee (record 11/LO/1924) and by the local research and development committees of University College London Hospitals, Great Ormond Street Hospital, and Guy's and St Thomas's NHS Foundation Trust. Patients or their legal guardians (as appropriate) were approached at the time of presurgical assessment by one of the research fellows (H.A.J., P.K.T.). Signed informed consent was obtained to allow the excised RV tissue to be retained for experimentation after the removal of residual excessive muscle bundles in the RV outflow tract. Consent was also obtained for the retrospective use of clinical data. To allow comparison with RV samples from nondiseased tissue, we also used frozen samples of RV tissue that had been obtained from 7 unused donor hearts; these were selected so that their sex and age profiles (4 male, 3 female; mean patient age, 28±13 years) resembled those of the patients with rToF undergoing PVR. These samples were obtained from the Sydney Heart Bank, Australia, with full consent of the patients' representatives and with ethics approval (protocol 2012/2814) from the Human Research Ethics Committee of the University of Sydney.

### Clinical Assessments

Eight patients (3 male, 5 female; mean patient age, 24±12 years) with previous ToF repair underwent PVR for increasing RV volumes associated with symptoms of shortness of breath on exertion and/or palpitations (median New York Heart Association [NYHA] class II) in agreement with current clinical indications. As part of their clinical preoperative assessment, all patients underwent echocardiogram, CMR, and cardiopulmonary exercise testing, as described below and previously.^13^

### Echocardiography

Standard Doppler echocardiography was performed with a VIVID 7 machine (GE Medical Systems) equipped with a multifrequency transducer (3.5 and 5 MHz), as described previously.^7^ From the apical view, tricuspid annular plane systolic excursion was obtained from M‐mode interrogation of the lateral aspect of the tricuspid valve. From the same apical views, tissue Doppler myocardial velocities were obtained at the RV and LV lateral annulus to assess longitudinal function. LV ejection fraction was calculated from the parasternal long‐axis view using the Simpson formula. The degree of tricuspid valve regurgitation was assessed qualitatively using color Doppler from the apical 4‐chamber view and from the short‐axis view; the regurgitation was graded as trivial, mild, moderate, or severe according to the Doppler intensity. From the tricuspid valve regurgitation jet, the RV systolic pressure was obtained using the Bernoulli equation. Peak velocity gradient across the RV outflow tract was calculated from the maximum velocity obtained from the continuous‐wave Doppler signal.^14^ Pulmonary valve regurgitation was assessed by color and pulsed Doppler interrogation of the pulmonary valve (pressure half time <100 ms) and of the main pulmonary artery and branch pulmonary arteries (presence of reversal flow).

### CMR Imaging

CMR was performed with a 1.5‐T magnetic resonance scanner (Avanto; Siemens Medical Systems) using techniques described previously.^7^ Assessment of LV and RV volumes was performed by manual segmentation of short‐axis cine images at end‐diastole and end‐systole (Argus; Siemens Medical Systems). End‐diastolic and end‐systolic volumes were calculated by use of the Simpson rule for each ventricle, and from these volumes, stroke volume and ejection fraction were calculated. Arterial blood flow was calculated from phase contrast images by use of a semiautomatic vessel edge‐detection algorithm (Argus; Siemens Medical Systems) with operator correction. Pulmonary valve regurgitant fraction was calculated as percentage of backward flow over forward flow. All volume and flow measurements were indexed for body surface area.

### Cardiopulmonary Exercise Testing

Cardiopulmonary exercise testing was performed on an electronically braked bicycle ergometer (ER 900, Ergoline) with respiratory gas exchange analysis. We used a ramp protocol, as described previously.^7^ Peak oxygen uptake, oxygen uptake at the anaerobic threshold, and ventilatory response to carbon dioxide production were derived from respiratory gas analysis during maximal exercise testing; ventilatory response to carbon dioxide production was measured as the slope for the whole exercise.

### Functional Studies of Human Permeabilized Cardiac Myocytes

We carried out functional studies of the myofilaments within human single permeabilized cardiac myocytes that had been prepared from rToF‐PVR tissue and RV donor tissue. Myofilament contractile function was assessed from passive myocyte stiffness (at <10 nmol/L Ca^2+^, which is below the threshold for Ca^2+^ activation of the myofilaments); force production of the myofilaments when maximally activated with Ca^2+^ (30 µmol/L Ca^2+^); maximum rate of force redevelopment after a brief release or restretch of the myocyte during maximal Ca^2+^ activation (which gives information on the rate of actomyosin crossbridge cycling); and Ca^2+^ sensitivity of myofilament force production, using a range of Ca^2+^ concentrations (0.1–30 µmol/L).

The permeabilized myocytes for these experiments were prepared at 4°C by a modification of the method used previously.^15^ A frozen piece of rToF‐PVR tissue or donor RV tissue was homogenized for 7 to 10 seconds in a 1‐mL ground‐glass hand homogenizer containing skinning solution (ie, relaxing solution with 1% Triton X‐100; ThermoFisher). Relaxing solution had the following composition (mmol/L): N,N‐bis(2‐hydroxyethyl)‐2‐aminoethanesulfonic acid (100), K propionate (55), Na_2_ phosphocreatine (10), Na_2_H_2_ATP (6.21; for MgATP^2−^=5), MgCl_2_ (6.24; for Mg^2+^=1), dithiothreitol (1), EGTA (10; to maintain the free Ca^2+^ concentration at ≈1 nmol/L; ‐log_10_[Ca^2+^] (pCa) ≈9.0), and protease inhibitors leupeptin (0.001), E64 (0.001), and AEBSF (0.25; all from Sigma‐Aldrich); pH was 7.1 at 4°C, and ionic strength was 0.20 mol/L. The homogenate in skinning solution was left on ice for 30 minutes for permeabilization of the myocyte membranes, then transferred into a Protein‐LoBind Eppendorf tube (Fisher Scientific) and centrifuged (1500 *g*, 4.5 minutes). The myocyte pellet was washed 3 times in relaxing solution (4000 rpm, 4.5 minutes each time) to remove the Triton X‐100. The final myocyte suspension in relaxing solution was then stored on ice.

The experimental protocol was modified slightly from that described previously.^15^ A permeabilized myocyte was selected, and its ends were glued with UV‐setting glue (OA63 adhesive and Opticure LED‐200 UV light source; Norland) between pins extending from a force transducer and a high‐speed length controller (403A and 315C‐I; Aurora Scientific). The mean sarcomere length (SL), measured using an Aurora 600A video analysis system in autocorrelation mode, was set to 2.0 μm. Myocytes were rejected if the cell was visibly damaged, their end attachments were unstable, the resting sarcomere pattern was unclear or misaligned, the force during Ca^2+^‐activated contraction was unstable, or the resting SL after a contraction was substantially different from that before contraction. These criteria, plus the fact that it was difficult to prepare more than a few myocytes from the small amounts of rToF‐PVR tissue available, meant that it was impossible to obtain useful data from the same number of myocytes from each patient sample. The numbers of data‐yielding myocytes from each donor or patient ranged from 1 to 6.

**Figure 1:**
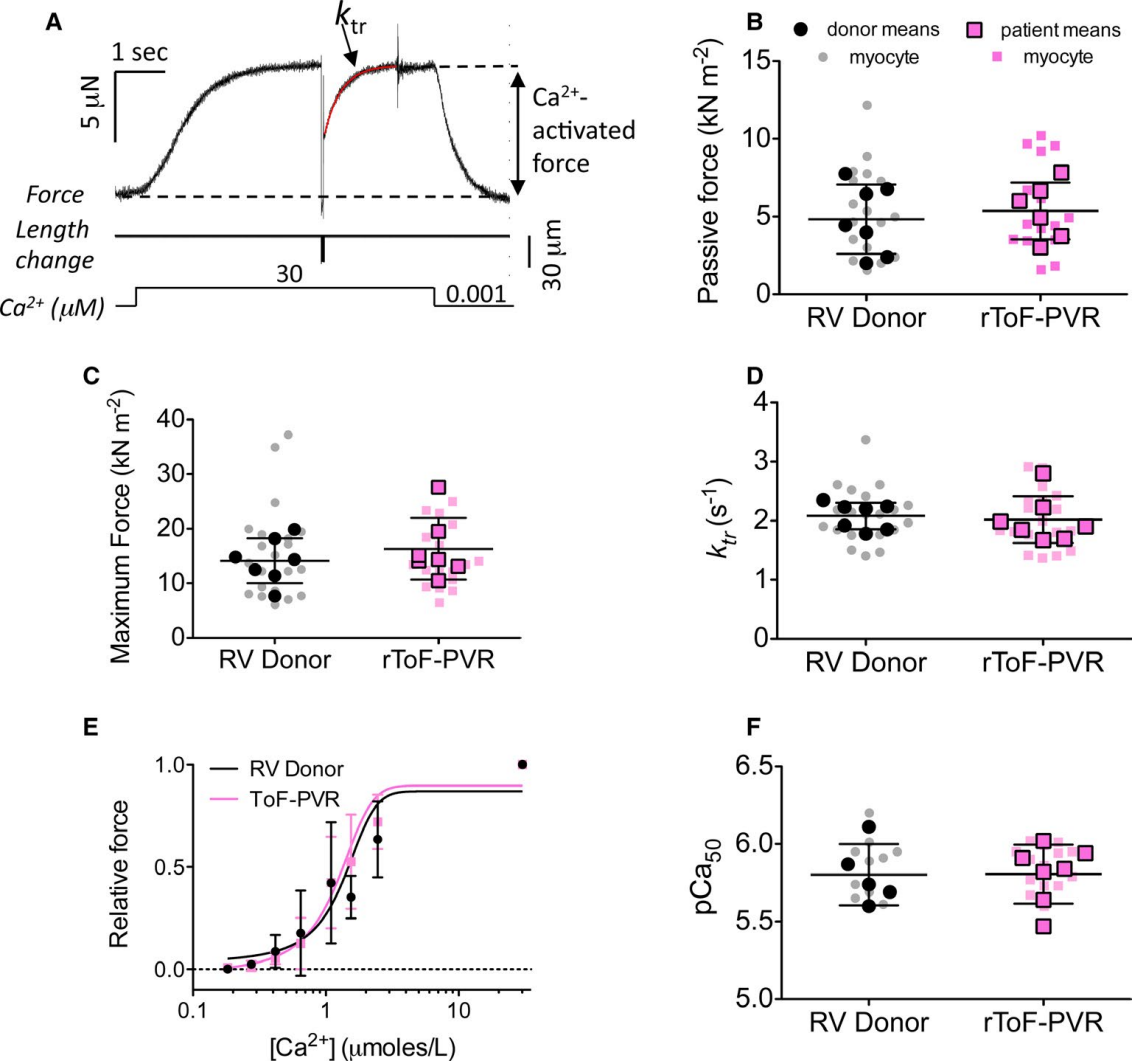
**Passive stiffness and Ca^2+^ activated force in “permeabilized” cardiac myocytes from patients with repair of tetralogy of Fallot undergoing pulmonary valve replacement (rToF‐PVR) are similar to those from right ventricle (RV) donors.** **A**, A typical force trace showing activation of a single myocyte by an increase in solution Ca^2+^ and the measurement of Ca^2+^‐activated force and crossbridge kinetics (*k*_tr_). For (**B**, **C**, **D**, and **F**), each small symbol shows the result from 1 myocyte, and each large symbol shows the mean derived from replicate myocytes from each donor or patient. The horizontal bars show the overall mean±SD, calculated using each mean value derived from replicate myocytes (large symbols) as a single datum for the statistical analysis. **B**, Passive force, measured as the difference between passive forces at sarcomere lengths 2.0 and 2.3 µm. There were 19 RV donor myocytes (1–6 myocytes from each of 7 donors used in this panel) and 16 rToF‐PVR myocytes (1–4 myocytes from each of 6 patients with rToF undergoing PVR used in this panel). **C**, Maximum Ca^2+^‐activated force, corrected for myocyte cross‐sectional area. There were 23 donor myocytes (1–6 myocytes from each of 7 donors) and 19 rToF‐PVR myocytes (1–4 myocytes from each of 7 patients). **D**, Rate of redevelopment of force (*k*_tr_) after a release/restretch protocol at maximum Ca^2+^. There were 21 donor myocytes (1–6 myocytes from each of 7 donors) and 19 rToF‐PVR myocytes (1–4 myocytes from each of 7 patients). **E**, Force–Ca^2+^ relationship. All forces are expressed relative to the maximum force at 30 µmol/L Ca^2+^. In this panel, the symbols show overall mean±SD for each [Ca^2+^]. There were 1 to 6 myocytes from each of 5 donors and 1 to 4 myocytes from each of 7 patients. **F**, Ca^2+^ sensitivity, expressed as the pCa50 value (−log_10_ of [Ca^2+^] required for 50% activation of force), and serves as a summary variable for the same data as in (**E**). Statistical analysis showed no significant differences (*P*>0.05, unpaired *t* tests) between the overall mean data for RV donor and patients with rToF undergoing PVR for any of the measured parameters.

To measure its passive stiffness, the permeabilized myocyte was bathed in relaxing solution at 15°C and given stretches of 1‐second duration and various magnitudes to increase SL from 2.0 µm to within the range of 2.1 to 2.3 µm.^15^ For this study, we measured the force increment at the end of a stretch from SL 2.0 to 2.3 µm and used this increase in resting force as a measure of myocyte passive stiffness.

To examine the Ca^2+^‐activated contractile performance of the permeabilized myocyte, we then perfused the myocyte with a series of CaEGTA‐containing activating solutions of Ca^2+^ 0.1 to 30 µmol/L (pCa 7.0–4.5; pH 7.1; 15°C).^15^ Once force had developed to a steady level (Figure 1A), the myocyte was subjected to a release/restretch protocol to detach all attached myosin crossbridges from actin. The subsequent recovery of force, as crossbridges reattached to actin, was fitted with a single exponential fit, and the rate constant of tension recovery (*k_tr_*) was used as a measure of crossbridge kinetics. Ca^2+^‐activated force was measured as the total steady force in activating solution minus the preceding passive force (at SL 2.0 µm). The relationship between Ca^2+^‐activated force and Ca^2+^ concentration of the activation solution was fitted with a sigmoidal curve (Hill equation) that was used to calculate the pCa required for 50% activation of force (pCa50).

The sensitivity of these techniques to detect functional changes in myocytes from diseased myocardium was validated in our previous studies, in which we found decreased maximum force but increased *k_tr_* and Ca^2+^ sensitivity in myocytes from hypertrophic cardiomyopathy patients^15^ and increased Ca^2+^ sensitivity in myocytes from a dilated cardiomyopathy patient.^16^

### Histologic Quantification of Collagen

Cryosections cut from frozen blocks of tissue were subjected to xylene clearance and sequential ethanol treatment. Slides were left in Milli‐Q H_2_O for 5 minutes followed by a 30‐second incubation in 0.2% phosphomolybdic acid. Slides were rinsed in Milli‐Q H_2_O and then left in 1% picrosirius red solution (to stain for collagen) for 90 minutes. Slides were washed 2×2 minutes in acidified Milli‐Q H_2_O (0.05% acetic acid), incubated for 15 minutes in picric acid, rinsed 3 times in Milli‐Q H_2_O, then dehydrated by sequential ethanol treatment (25%, 50%, 75%, 96% [1 minute each]; 100% [2×3 minutes]; xylene [2×5 minutes]). Glass coverslips were mounted with DPX (Sigma‐Aldrich) and allowed to dry overnight. Sections were viewed with a light microscope (Zeiss), without and with a polarization filter, to identify collagen organization. Images were captured and analyzed using Volocity software (Perkin Elmer).

### Microarray Gene Expression Profiling

RNA extraction was performed on LN_2_ cooled and pulverized heart tissue samples using the Direct‐zol RNA Miniprep Plus Kit (Zymo Research Corp), according to the manufacturer's instructions. RNA quality was assessed using the Agilent 2100 bioanalyzer (Agilent Technologies) and quantified using the Nanodrop ND‐1000 spectrophotometer (Thermo Fisher Scientific). All 7 rToF‐PVR and 6 of 7 RV donor samples passed quality control and were subsequently subjected to microarray analysis. The gene expression profiles were determined using the GeneChip Human Transcriptome 2.0 Array (Affymetrix; ThermoFisher Scientific). Single‐primer isothermal amplified cDNA was generated using the Ovation Pico WTA System V2 Kit (Nugen; AC Leek), following the manufacturer's instructions. In addition, the single‐primer isothermal amplified cDNA was subjected to a quality‐control check to assess quality (Agilent 2100 bioanalyzer) and quantity (Nanodrop ND‐1000 spectrophotometer) in preparation for the next stage. The single‐primer isothermal amplified cDNA was fragmented and biotin‐labeled using the Encore Biotin Module (Nugen), according to the manufacturer's instructions. The fragmented and biotin‐labeled cDNA was subjected to a further round of quality‐control checks to assess fragmentation size (Agilent 2100 bioanalyzer). Hybridization cocktails were prepared from the fragmented labeled cDNA according to Nugen's recommendations and hybridized to the microarrays at 45°C for 18 hours. The arrays were washed and stained using the wash protocol FS450_0001 recommended for GeneChip Human Transcriptome 2.0 arrays on the GeneChip Fluidics 450 station. The arrays were scanned using the GeneChip Scanner 3000 7G. CEL files were assessed for quality control in the Expression Console software package (Affymetrix; ThermoFisher Scientific) using standard metrics and guidelines for the Affymetrix microarray system. Principal component analysis, hierarchical clustering, and gene set enrichment analysis were performed in Qlucore Omics Explorer 3.0 (Qlucore, Lund, Sweden). Alignment and comparison to the Gene Ontology (GO) database of biological processes was performed in MetaCore (Thompson Reuters) and used to identify processes and pathways represented by the differentially regulated genes. These microarray data have been submitted to NCBI GEO and are accessible through accession number GSE141955.

### Quantitative Polymerase Chain Reaction

Using 2 μg of RNA from the above extraction process, cDNA was synthesized using an RT^2^ First‐Strand Kit (Qiagen). Quantitative polymerase chain reaction (PCR) was carried out using the ΔCt method. To start, 9 μL of cDNA was added to 10 μL 2x Sybr Green PCR Master Mix and 5 μmol of forward and reverse primers for the protein of interest (Table 1). Cycling parameters were 94°C for 15 seconds, followed by single‐step annealing and extension at 60°C for 1 minute (35 cycles). Reactions were carried out in a Corbett RotorGene‐3000. The cycle threshold was determined automatically by the software and corresponds to a point during the linear phase of amplification. Gene expression of rToF‐PVR samples was expressed as fold change compared with RV donor samples by normalizing individual data points to the mean of RV donors (defined as the control group), which fixed the mean value for the RV donor group to 1.

**Table 1:** Primers Used in This Investigation for Quantitative PCR

Gene	Forward	Reverse
*ASPN*	5′‐CTC TGC CAA ACC CTT CTT TAG C‐3′	5′‐CGT GAA TAG CAC TGA CAT CCA A‐3′
*LUM*	5′‐TAA CTG CCC TGA AAG CTA CCC‐3′	5′‐GGA GGC ACC ATT GGT ACA CTT‐3′
*OGN*	5′‐TCT ACA CTT CTC CTG TTA CTG CT‐3′	5′‐GAG GTA ATG GTG TTA TTG CCT CA‐3′
*COL1A2*	5′‐GAG CGG TAA CAA GGG TGA GC‐3′	5′‐CAC CCT GTG GTC CAA CAA CTC‐3′
*COL3A1*	5′‐GGA GCT GGC TAC TTC TCG C‐3′	5′‐GGG AAC ATC CTC CTT CAA CAG‐3′
*LOX*	5′‐GCC GAC CAA GAT ATT CCT GGG‐3′	5′‐GCA GGT CAT AGT GGC TAA ACT C‐3′
*GAPDH*	5′‐GGA GCG AGA TCC CTC CAA AAT‐3′	5′‐GGC TGT TGT CAT ACT TCT CAT GG‐3′

### Extraction of ECM Proteins

Tissue was washed in PBS and then placed in 0.5 mol/L NaCl extraction buffer (1:10 wt/vol) and shaken (at low speed) for 1 hour at room temperature (RT). Decellularization was then performed in 0.1% SDS, with 25 mmol/L EDTA and a 1:100 cocktail of protease inhibitors (1:10 wt/vol) and shaken for 18 hours at RT. Finally, samples were added to 4 mol/L guanidine‐HCl (1:5 wt/vol) extraction buffer and shaken vigorously for 48 hours at RT to extract the strongly bound ECM components. Guanidine extracts containing 25 μg of protein were subjected to ethanol precipitation at −20°C overnight after adding 10x the volume of ethanol to the extract lysate. Samples were centrifuged at 14 000*g* for 45 minutes at 4°C. The supernatants were removed, and the protein‐containing pellets were centrifuged in a vacuum centrifuge for 30 minutes at 37°C to dry the pellet completely. The pellets were then subjected to complete deglycosylation to remove any glycosaminoglycan chains or N‐ and O‐linked oligosaccharides that might interfere with antibody binding. Deglycosylation was performed by adding to the pellet a deglycosylation buffer (150 mmol/L NaCl, 50 mmol/L sodium acetate [pH 6.8], 10 mmol/L EDTA, and supplemented with protease inhibitors) with enzymes: heparinase (1:500), chondroitinase ABC (1:100), keratinase (1:500; all Sigma‐Aldrich); PNGaseF (1:200) and 3 different debranching enzymes (α2‐3,6,8,9‐neuraminidase [1:200], ß‐N‐acetylglucosaminidase [1:200], and O‐glycosidase for complete removal of O‐linked sugars (1:200; all from Millipore). These were incubated with shaking for 48 hours at 37°C. Sample buffer containing β‐mercaptoethanol was added to the deglycosylated samples to a concentration of 0.5 μg/μL in preparation for western blotting.

### Western Blotting

Deglycosylated lysates were heated to 85°C for 10 minutes. A chamber was prepared with a gradient SDS gel of 4% to 16% acrylamide concentration. Next, 5 μg of 0.5‐μg/μL samples were loaded into each well of the gel. The proteins were separated at 175 mV. Transfer was achieved in wet conditions. The proteins were electrophoretically transferred onto nitrocellulose membranes using 350 mA at RT for 2 hours. Membranes were blocked for 1 hour with 5% milk in PBS with Tween. Primary antibodies were added with PBS with Tween/5% BSA and incubated overnight at 4°C. The membrane was washed 3×15 minutes with PBS with Tween. The blots were then incubated in secondary antibody conjugated to horseradish peroxidase for 1 hour. The blots were again washed 3×15 minutes with PBS with Tween, and bound antibody was detected by enhanced chemiluminescence (ECL; GE Healthcare) onto film. Images were scanned and imported to Image Studio Lite (Li‐Cor Biosciences) for densitometric analysis.

### Immunofluorescence Staining

Tissue was removed from storage at −80°C, warmed to −20°C for ≈1 hour, then mounted onto the stage of a cryostat with OCT compound and allowed to equilibrate to the ambient temperature of the cryostat for 5 minutes. For cutting tissue, the stage temperature was set to −22°C, and the knife was set to −20°C. The sections were then cut to 10 μm thick and mounted onto high‐quality Superfrost slides (Thermo Fisher Scientific). The sections were allowed to air dry for ≈1 hour and then stored at −80°C. For staining, sections were allowed to dry completely and fixed by immersion, in a Coplin jar, in precooled 100% methanol at −20°C for 5 minutes or 4% formalin for 10 minutes at RT. Sections were washed 2×5 minutes in PBS, then permeabilized in 0.5% NP‐40 for 3 minutes at RT and washed 3×5 minutes in PBS. Sections were blocked for 1 hour at RT in 5% donkey serum. Primary antibody was diluted appropriately in blocking solution, applied to the sections, and incubated in a humidified chamber overnight at 4°C. Sections were washed 3×5 minutes in PBS, and secondary antibody conjugated to a fluorophore was diluted as appropriate, applied to the sections, and incubated in the dark in a humidified chamber for 1 hour at RT. DAPI was added 1:10 000 for 5 minutes at the end of the incubation for visualization of nucleic structures. Sections were washed 3×5 minutes in PBS in the dark and then mounted in Mowiol mounting media (Kuraray Specialties europe Gmbh) and allowed to dry in the dark overnight. Images were acquired with an A1R point‐scanning confocal microscope (Nikon).

### Antibodies

The following antibodies were used in this investigation: antiasporin (Thermo Fisher [PA5‐13553]; polyclonal rabbit IgG, diluted 1:1000 for western blotting and 1:100 for immunofluorescence); antilumican (Santa Cruz [sc‐33785]; polyclonal rabbit IgG, diluted 1:100 for western blotting); antimimecan/osteoglycin (Abcam [ab110558]; polyclonal rabbit IgG, diluted 1:1000 for western blotting); antimyomesin (gift from Elisabeth Ehler; mouse monoclonal IgG, clone B4 diluted 1:100 for immunofluorescence). In the case of asporin, for which band specificity was difficult to determine, we performed sequence alignment of the epitope and discovered homology to biglycan, which has predicted a molecular weight ≈5 kDa smaller than asporin; therefore, we believe the lower band visible on the membrane identifies biglycan. We ascribe the higher band to incomplete removal of glycosaminoglycans.

### Statistical Analysis

Results of clinical investigations (CMR, cardiopulmonary exercise testing, and echocardiography) were expressed as mean±SD. Data from functional experiments, quantitative PCR experiments, and image analysis of picrosirius red staining were plotted individually and expressed as mean±SD. As appropriate, the unpaired Student *t* test was applied to test for differences between rToF‐PVR and RV donor groups. For the functional data (force, *k*_tr_, pCa required for 50% activation of force), the data from multiple myocytes were averaged for each patient and the mean value for each patient was taken as a single datum point for the subsequent calculation of overall mean and SD and *t* tests. Because of the technically challenging nature of the work, the number of myocytes per patient or donor was variable (range, 1–6). If distribution of the data were not normal, the Mann–Whitney test was used, whereas the Welch correction was applied when the variance was unequal between data sets. For transcriptome (mRNA) analysis, 2‐group comparison with *t* test was performed, and a threshold *P* value of 0.004 and a false‐discovery rate of 0.47 were selected for principal component analysis and hierarchical clustering. For GO enrichment, a hypergeometric test was used with *P*<0.05 adjusted by Benjamini‐Hochberg correction.

## Results

### Patient Characteristics

Eight patients underwent PVR, with a mean age of 24±12 years (range, 11–49 years). Seven of 8 patients had a transannular patch at the time of initial rToF and 2 of 8 patients were previously palliated with a right modified Blalock–Taussig shunt. One patient had initial rToF with valve preservation (age, 3 months) and required subsequent balloon valvotomy (age, 9 months) and redo repair with transannular patch (age, 10 months). Only 2 patients had associated comorbidities; 1 patient (age, 15 years at the time of PVR) had mild right hemiplegia as result of an embolic event at the time of primary repair and some hearing impairment, whereas another patient (age, 11 years at the time of PVR) had a history of a high level of consanguinity and presented with intrauterine growth restriction with associated learning difficulties, bilateral Perthes disease, and eczema. Only 1 patient (age, 49 years) was on medication before PVR; medications included β‐blocker (bisoprolol 1.25 mg), aspirin 75 mg, and diuretics given only a few weeks before surgery (furosemide 20 mg and spironolactone 25 mg). Patient characteristics are summarized in Table 2. All patients had severe pulmonary valve regurgitation (pulmonary valve regurgitant fraction, 48±7%), whereas there was no evidence of significant residual RV outflow tract obstruction (mean RV outflow tract peak velocity, 2.2±0.4 m/s). The RV was significantly dilated, with an RV end‐diastolic volume indexed for body surface area of 149±26 mL/m^2^ and a ratio of 2.2±0.4 for RV:LV end‐diastolic volume indexed for body surface area but with preserved systolic function (RV ejection fraction, 56±5%). LV volumes and systolic function were normal in all (mean LV end‐diastolic volume indexed for body surface area, 71±19 mL/m^2^; mean ejection fraction, 66±7%). Two patients had moderate tricuspid valve regurgitation, 1 patient had mild tricuspid valve regurgitation, and the others had trivial or no regurgitation. On cardiopulmonary exercise testing, exercise capacity was found to be reduced only mildly: mean peak oxygen uptake measured 31±8 mL/min per kg (84±11% of predicted) with a slope for ventilatory response to carbon dioxide production of 28±4.

**Table 2:** Patient Characteristics: Demographic, TTE, CPET, and CMR Imaging Data

	Patients (n=8)
Male:female ratio	3:5
Age at repair, y	2±2.6
Age at PVR, y	24±12
NYHA class, n	
I	2
II	6
TTE	
TAPSE, mm	21±4
TR velocity, m/s	2.5±0.4
RVOT peak velocity, m/s	2.2±0.4
CPET	
Peak Vo_2_, mL/min/kg	31±8
Peak Vo_2_ % of predicted	84±11
VE/Vco_2_ slope	28±4
Peak HR, beats/min	180±13
CMR	
RV EDVi, mL/m^2^	149±26
RV ESVi, mL/m^2^	65±17
RV:LV EDV ratio	2.2±0.4:1
RV CO, L/m^2^	6.3±1.8
RV EF, %	56±6
LV EDVi, mL/m^2^	71±19
LV EF, %	66±7
LV CO, L/m^2^	3.5±0.4
PV RF, %	48±7

CMR indicates cardiac magnetic resonance; CO, cardiac output; CPET, cardiopulmonary exercise testing; EDV, end‐diastolic volume; EDVi, end‐diastolic volume indexed for body surface area; EF, ejection fraction; ESVi, end‐systolic volume indexed for body surface area; HR, heart rate; LV, left ventricle; NYHA, New York Heart Association; PV RF, pulmonary valve regurgitant fraction; PVR, pulmonary valve replacement; RV, right ventricle; TAPSE, tricuspid annular systolic excursion; TR, tricuspid valve regurgitation; TTE, transthoracic echocardiogram; VE/Vco_2_, ventilatory response to carbon dioxide production; and Vo_2_, oxygen uptake.

Nondiseased RV tissue samples from 7 unused donor hearts were used as a reference in this study. The donors were selected so that their sex and age profiles (4 male, 3 female; patient age, 28±13 years) resembled those of the patients with rToF undergoing PVR. Five of the 7 donors had a subarachnoid hemorrhage after a motor vehicle accident, 1 donor died from a basal infarct after a grand mal seizure, and 1 donor died from hypoxia secondary to a seizure. Before explant, the hearts were classified as “healthy” but were not used for transplantation owing to lack of a tissue‐type match. LV or RV tissue from the same hearts has been used as reference tissue in previous publications by our and other laboratories.^15^, ^17^, ^18^, ^19^, ^20^, ^21^

### Stiffness and Contractile Function Were Similar for Permeabilized rToF‐PVR and RV Donor Myocytes

Previous studies have found that in left or right heart failure, there are changes in the passive stiffness and Ca^2+^‐activated force development of the cardiac myofilaments (see Discussion); in vivo, these alterations would be expected to contribute to diastolic and systolic dysfunction, respectively. We investigated whether similar changes could be observed with the myofilaments from the RVs of the patients with rToF undergoing PVR by measuring force development in permeabilized myocytes (Figure 1). The donor and rToF‐PVR permeabilized myocytes used in the functional experiments had similar cross‐sectional areas: 395±66 µm^2^ versus 411±57 µm^2^ for donor and rToF‐PVR myocytes, respectively. For each myocyte, we measured the passive stiffness of the myofilaments, as assessed by the increase in passive force on sarcomere stretch from 2.0 to 2.3 µm. There was some variability between the passive stiffnesses of the permeabilized myocytes within and between patient samples, but overall there was no significant difference in passive stiffness between the rToF‐PVR and donor myocytes (Figure 1B), with passive forces of 5.36±1.82 kN·m^−2^ (n=6 patients) and 4.83±2.23 kN·m^−2^ (n=7 donors; *P*=0.65, unpaired *t* test of patients versus donors), respectively.

Following the passive stiffness measurements, the myocytes were activated with Ca^2+^, and their Ca^2+^‐activated contractile properties were studied for the following key parameters: maximum Ca^2+^‐activated force (Figure 1C) was 16.3±5.6 kN·m^−2^ in patients with rToF undergoing PVR (n=7 patients) and 14.1±4.1 kN·m^−2^ in RV donors (n=7 donors; *P*=0.42). Crossbridge cycling kinetics, *k*_tr_, at maximal Ca^2+^ (Figure 1D) were 2.01±0.39 s^−1^ (n=7 patients) and 2.08±0.23 s^−1^ (n=7 donors; *P*=0.70). The Ca^2+^ sensitivity of force development (Figure 1E), as quantified by the pCa required for 50% activation of force (Figure 1F), was 5.80±0.19 (n=7 patients) and 5.80±0.20 (n=5 donors; *P*=0.97). Consequently, none of these resting or Ca^2+^‐activated contractile parameters showed a difference between patients with rToF undergoing PVR and donors. From these results, we concluded that the contractile function of myofibrils in permeabilized myocytes from the patients with rToF undergoing PVR was similar to that in donor myocytes.

### Collagen Content Was Similar in rToF‐PVR and RV Donor Tissue

**Figure 2:**
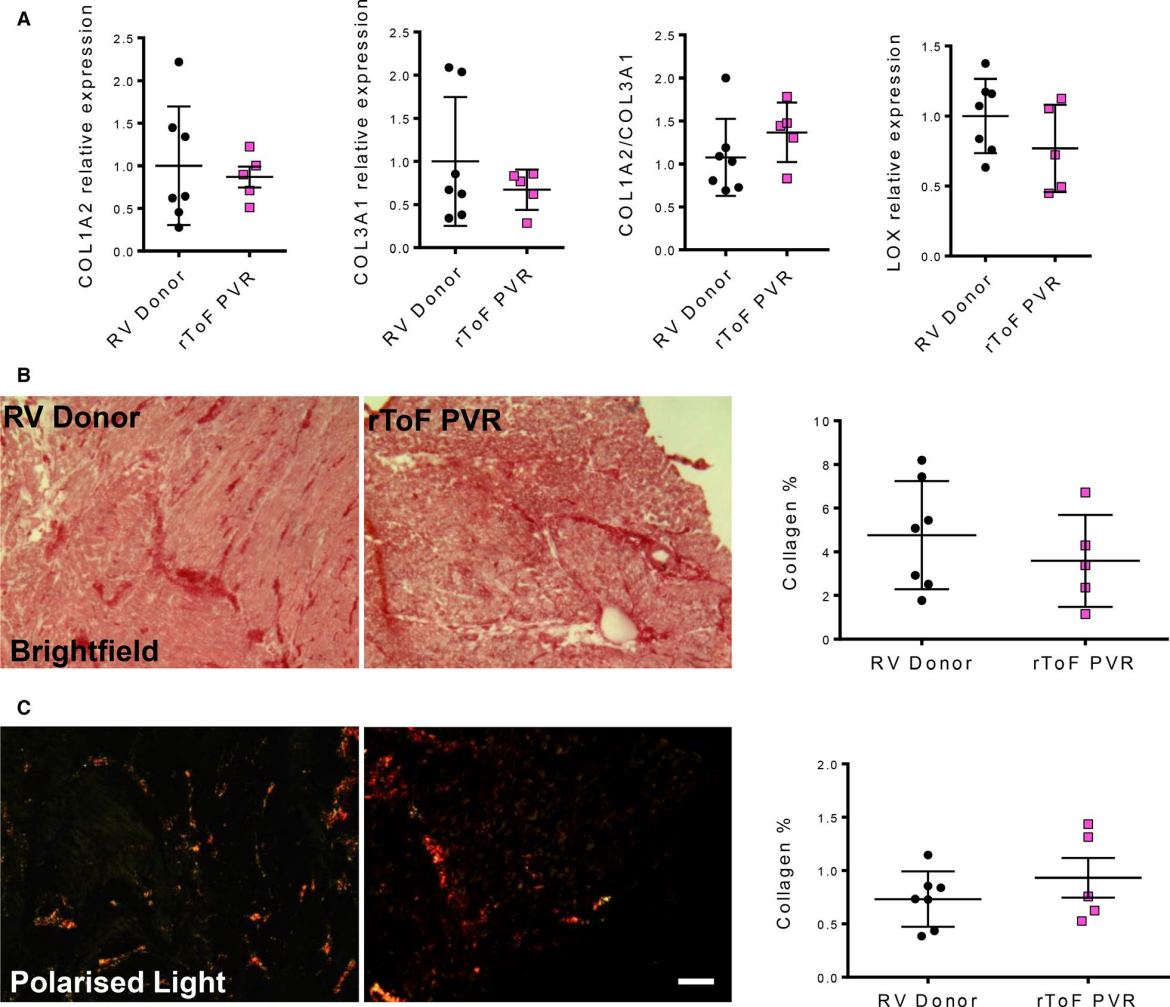
**The collagen matrix in myocardium of patients with repair of tetralogy of Fallot undergoing pulmonary valve replacement (rToF‐PVR) is similar to that in right ventricle (RV) donor myocardium.** **A**, quantitative polymerase chain reaction analysis of mRNA expression for COL1A2 (collagen I), COL3A1 (collagen III), and LOX (lysyl oxidase) indicated no differences between rToF‐PVR and RV donor myocardium. Each data point shows the result from 1 donor or rToF‐PVR patient. **B**, Picrosirius red staining of collagen fibers in RV donor and rToF‐PVR heart tissue sections with quantitation of the dark‐red‐stained collagen fibers as a percentage of total tissue area. **C**, The same sections were viewed under polarized light to assess the presence of irreversibly linked collagen as a percentage of total tissue area. No statistical differences were observed between groups for all assays described. RV donor, n=7; rToF‐PVR, n=5. Values are expressed as mean±SD. Scale=30 μm.

Collagens I and III are the major ECM protein constituents in the heart and, together with lysyl oxidase, which cross‐links the collagen, are known to be the main determinants of tissue stiffness.^22^ Quantitative PCR for mRNA expression of genes coding for collagen I, collagen III, and lysyl oxidase (*COL1A2*, *COL3A1*, and *LOX*, respectively) revealed that rToF‐PVR tissue was comparable with RV donor tissue (Figure 2A). Direct assessment of tissue structure by picrosirius red showed no quantifiable difference between the collagen content of rToF‐PVR and RV donor tissue under either bright‐field or circular polarized light (Figure 2B and 2C). The latter was used to visualize only the highly birefringent fibers and to identify potential deleterious organization of collagen that occurs as the fibrotic scar matures. In this setting, only the birefringent (bright) fibers are detected and indicate a predominance of collagen I over collagen III.^23^

### Transcriptomic Profiling Revealed Upregulation of Small Leucine‐Rich Proteoglycans in rToF‐PVR

**Figure 3:**
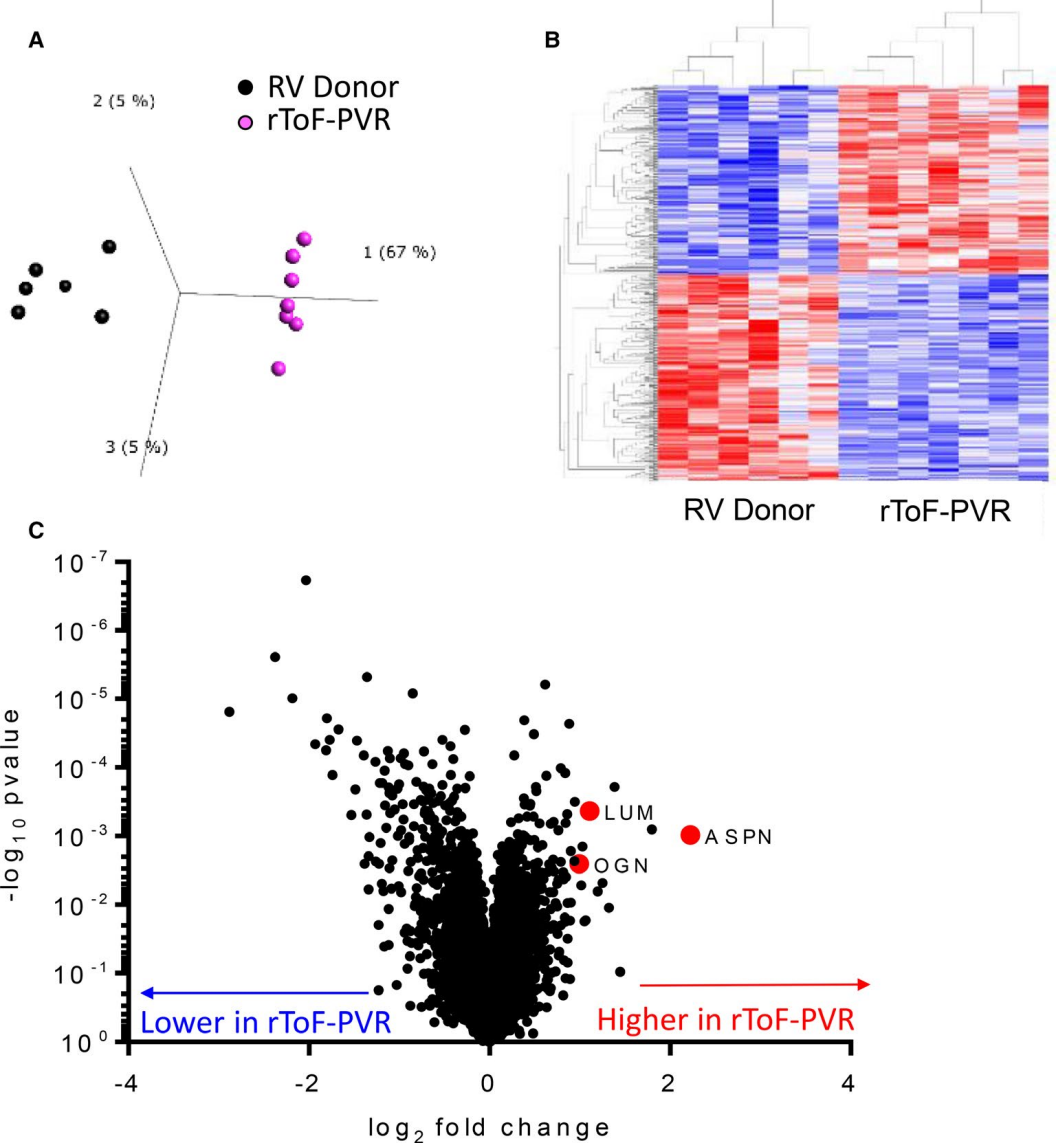
**Analysis of the transcriptome identifies gene‐expression changes in myocardium of patients with repair of tetralogy of Fallot undergoing pulmonary valve replacement (rToF‐PVR).** **A**, Principal component analysis of the gene‐expression profile of each tissue sample in relation to all others in the first 3 principal components revealed clear functional grouping of samples according to right ventricle (RV) donor (black) and rToF‐PVR (magenta). Each data point shows the result from 1 RV donor or rToF‐PVR patient. **B**, Hierarchical clustering also showed rToF‐PVR samples were functionally grouped according to gene‐expression profile. Selected thresholds were a false‐discovery rate (q) of 0.47 and *P*=0.004. **C**, Volcano analysis revealed that the most profound differences occurred in downregulated genes. The transcripts *ASPN*, *LUM*, and *OGN* were upregulated in rToF‐PVR samples. Statistical measures applied to the data set before analysis were a *P* value of 0.004 and a false‐discovery rate of 0.47. RV donor, n=6; rToF‐PVR, n=7.

**Figure 4:**
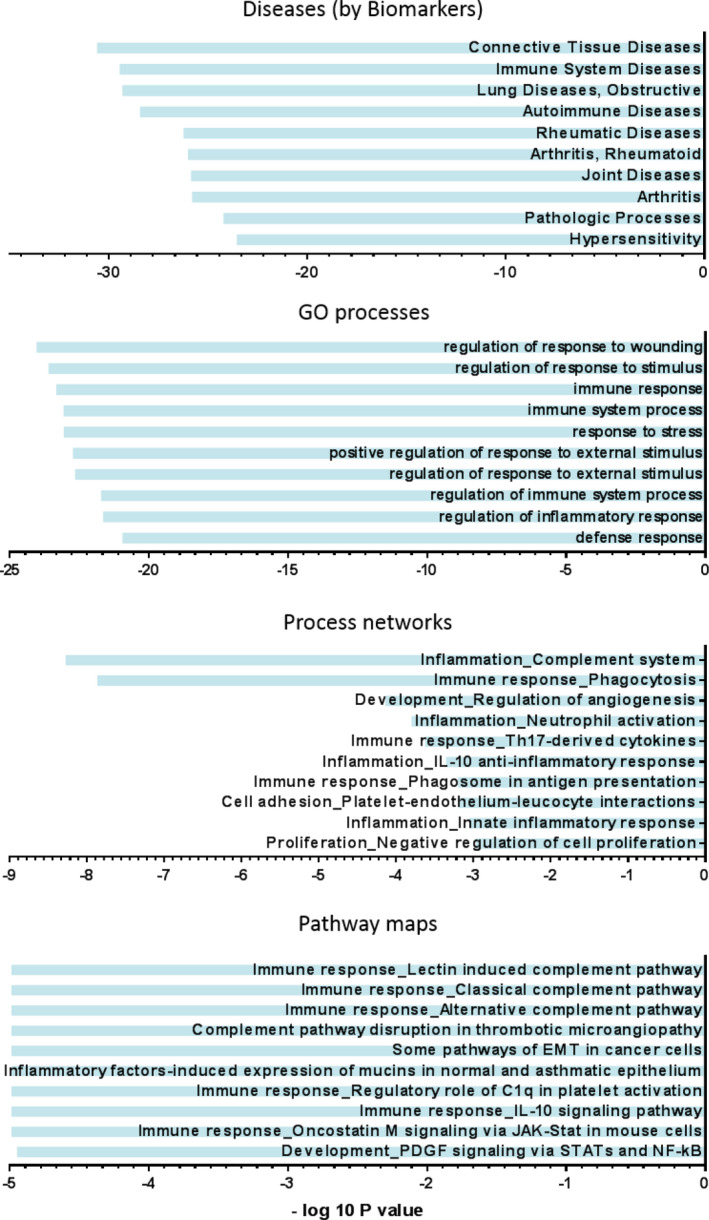
**Gene ontology (GO) analysis implicated tissue remodeling and inflammation pathways as dysregulated in myocardium of patients with repair of tetralogy of Fallot undergoing pulmonary valve replacement (rToF‐PVR).** Holistic analysis of the differentially expressed genes in rToF‐PVR myocardium compared with right ventricle donor against the GO‐annotated database of affected biological processes ordered according to *P* value.

**Figure 5:**
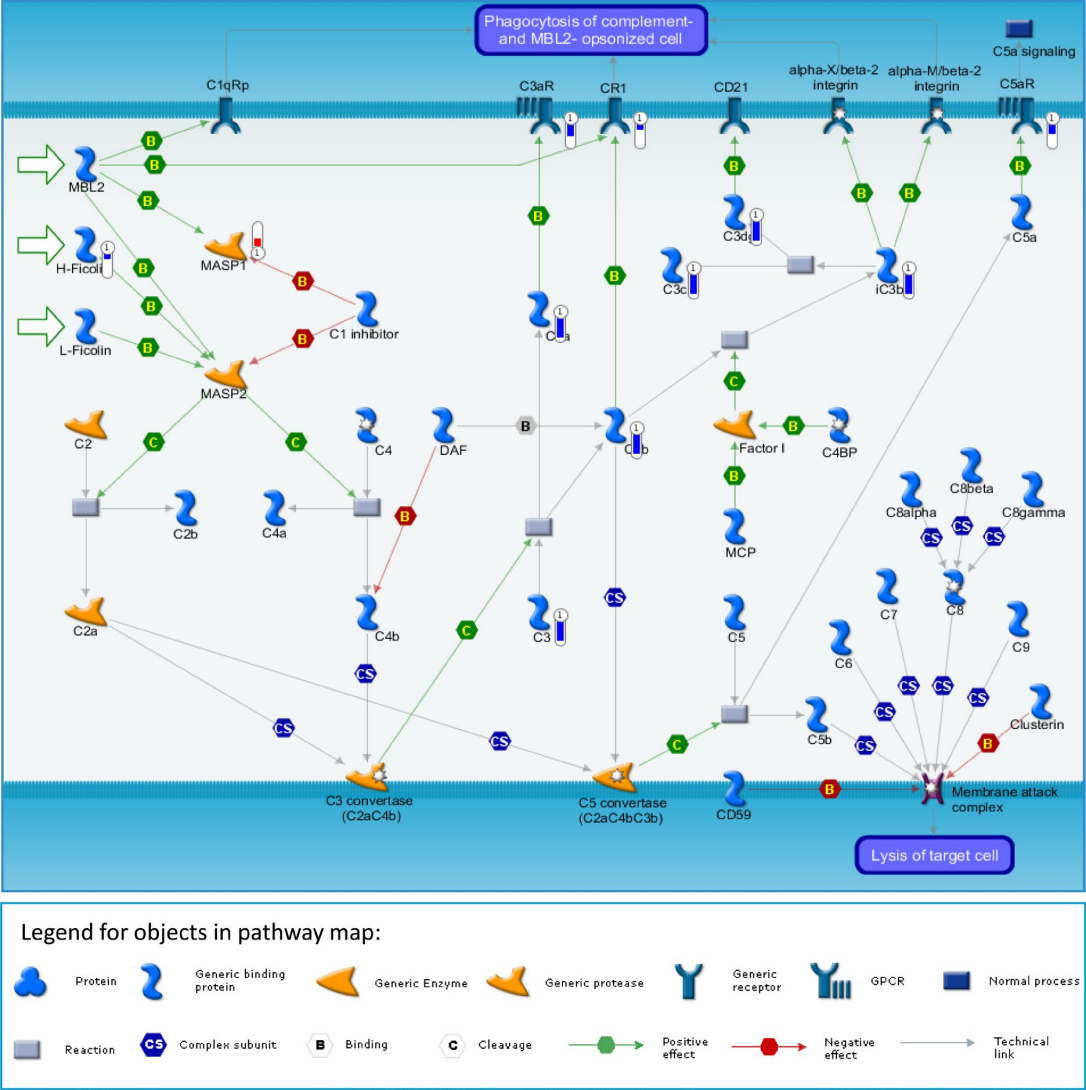
**Schematic representation of the top‐scoring, statistically significant pathway map indicated downregulation of genes involved in immune response in myocardium of patients with repair of tetralogy of Fallot undergoing pulmonary valve replacement** **(rToF‐PVR)** **.** The top‐scoring map (map with the lowest *P* value) based on enrichment distribution sorted by "statistically significant maps" was the Immune response_Lectin induced complement pathway. This shows negative regulation (blue thermometer) of most genes involved in this pathway, implying suppression of immune pathways in rToF‐PVR myocardium. Only 1 gene displayed upregulated expression (red thermometer). Many genes in the second and third top‐scoring maps overlapped with this map. The material in this figure is reproduced under a licence from Clarivate Analytics. This material may not be copied or redistributed in whole or in part without the written consent of Clarivate Analytics.

An Affymetrix human microarray was used to profile gene‐expression differences between heart tissue of patients with rToF undergoing PVR and RV donors. Principal component analysis, a multivariate analysis that places individual samples in 3‐dimensional space relative to each other based on false‐discovery rate (q) and *P* value, was performed. Application of thresholds (q=0.47 and *P*=0.004) showed clear clustering of samples within groups and clear separation between rToF‐PVR and RV donor samples (Figure 3A) and yielded a list of 601 transcripts, of which 330 encoded known genes (Table S1). Hierarchical clustering showed grouping by disease, with consistent groups of up‐ and downregulated genes that were evenly distributed (Figure 3B). Volcano analysis showed that the differentially regulated genes carrying the greatest degree of statistical significance were downregulated in rToF‐PVR myocardium (Figure 3C). GO pathway analysis clusters the differentially regulated transcripts by utilizing databases of gene names (or "ontologies") based on known associations as found in the existing body of literature. Using this method, we determined that "connective tissue diseases," "response to wounding," "inflammation," and "immune response" were the top scoring terms (ie, ones with the lowest *P* value) within their categories (diseases by biomarkers, GO processes, process networks, and pathway maps, respectively; Figure 4). Visualization of the top pathway map—immune response_Lectin induced complement pathway—showed that the transcripts in these pathways were predominantly downregulated (Figure 5).

**Figure 6:**
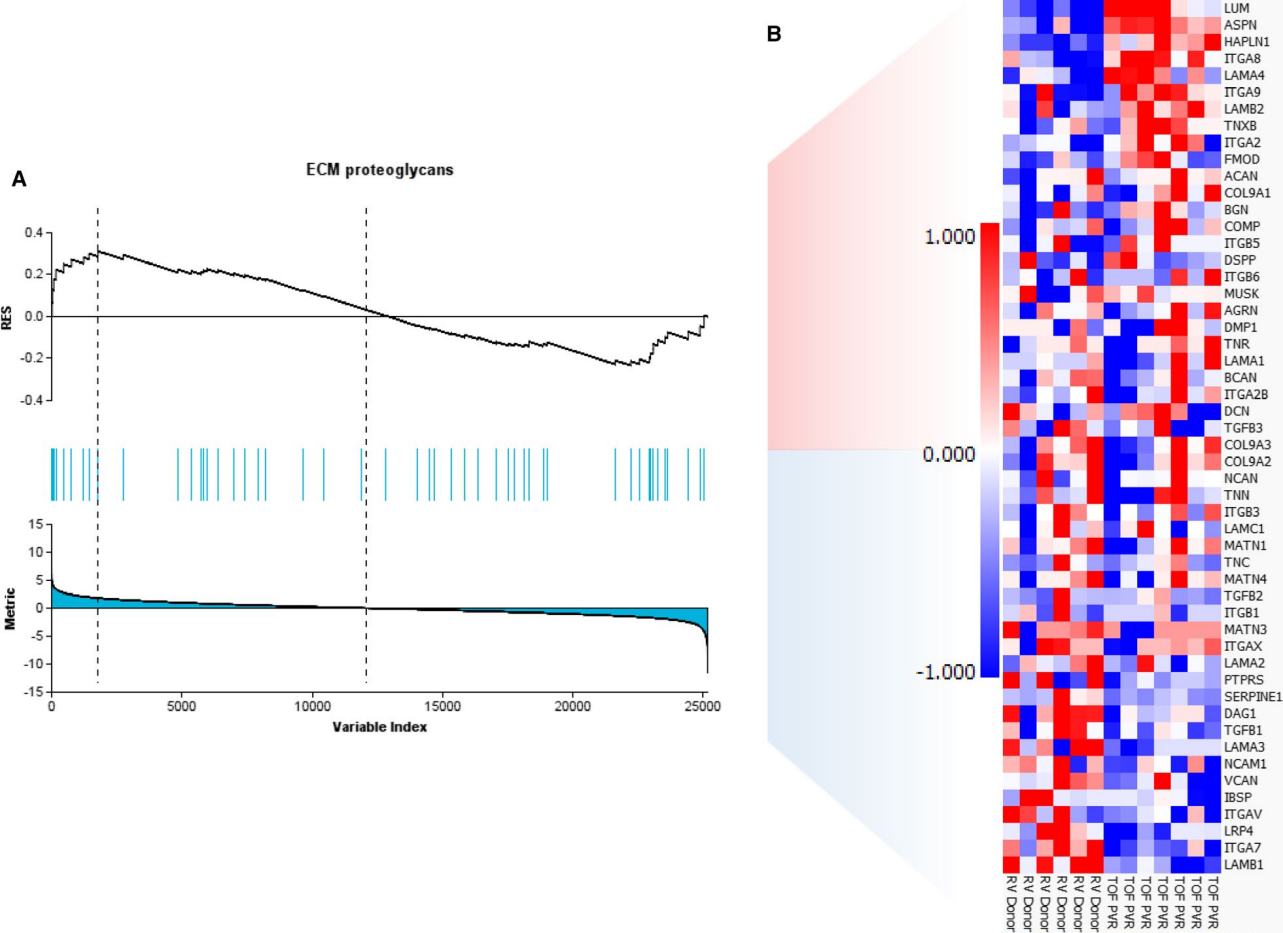
**Gene set enrichment analysis showing the expression patterns of the extracellular matrix (ECM) proteoglycan gene set in patients with repair of tetralogy of Fallot undergoing pulmonary valve replacement (rToF‐PVR) compared with right ventricle (RV) donor myocardium.** **A**, Gene set enrichment analysis of extracellular matrix proteoglycans displaying some enrichment of genes at the leading edge. **B**, Corresponding heat map of genes and expression profiles for 6 RV donor samples and 7 rToF‐PVR samples.

Lists of the 10 most up‐ and downregulated transcripts according to fold change (Table 3) showed that genes encoding asporin, lumican, and osteoglycin, which are all members of the small leucine‐rich proteoglycans (SLRP) family of ECM proteins, were upregulated in rToF‐PVR heart tissue compared with RV donor tissue. Gene set enrichment analysis showed that "ECM proteoglycans" displayed a modest enrichment of genes at the leading edges of the analysis, and transcripts were equally distributed between up‐ and downregulation (Figure 6).

**Table 3:** The 10 Most Upregulated Transcripts and 10 Most Downregulated Protein Coding Transcripts

Gene Name	Gene Symbol	Difference	Fold Change
Upregulated transcripts			
Asporin	*ASPN*	2.222	4.666
Guanylate cyclase activator 1C	*GUCA1C*	1.799	3.481
Frizzled‐related protein	*FRZB*	1.383	2.608
Lumican	*LUM*	1.108	2.156
Natriuretic peptide receptor C/guanylate cyclase C (atrionatriuretic peptide receptor C)	*NPR3*	1.027	2.038
Osteoglycin	*OGN*	0.9933	1.990
Crystallin, mu	*CRYM*	0.9442	1.924
Fc fragment of IgE, high affinity I, receptor for; alpha polypeptide	*FCER1A*	0.899	1.865
Fibronectin type III domain containing 1	*FNDC1*	0.881	1.842
Endothelin receptor type A	*EDNRA*	0.845	1.797
Downregulated transcripts			
RAS, dexamethasone‐induced 1	*RASD1*	−1.672	0.313
V‐set and immunoglobulin domain containing 4	*VSIG4*	−1.738	0.299
FK506 binding protein 5	*FKBP5*	−1.770	0.293
S100 calcium binding protein A9	*S100A9*	−1.800	0.287
Coagulation factor XIII, A1 polypeptide	*F13A1*	−1.811	0.284
Lymphatic vessel endothelial hyaluronan receptor 1	*LYVE1*	−1.929	0.262
Phospholipase A2, group IIA (platelets, synovial fluid)	*PLA2G2A*	−2.035	0.243
Serpin peptidase inhibitor, clade A (alpha‐1 antiproteinase, antitrypsin), member 3	*SERPINA3*	−2.183	0.220
CD163 molecule	*CD163*	−2.373	0.192
Metallothionein 1A	*MT1A*	−2.882	0.135

### SLRP Gene Expression Is Not Corroborated by Protein Abundance in rToF‐PVR Myocardium

**Figure 7:**
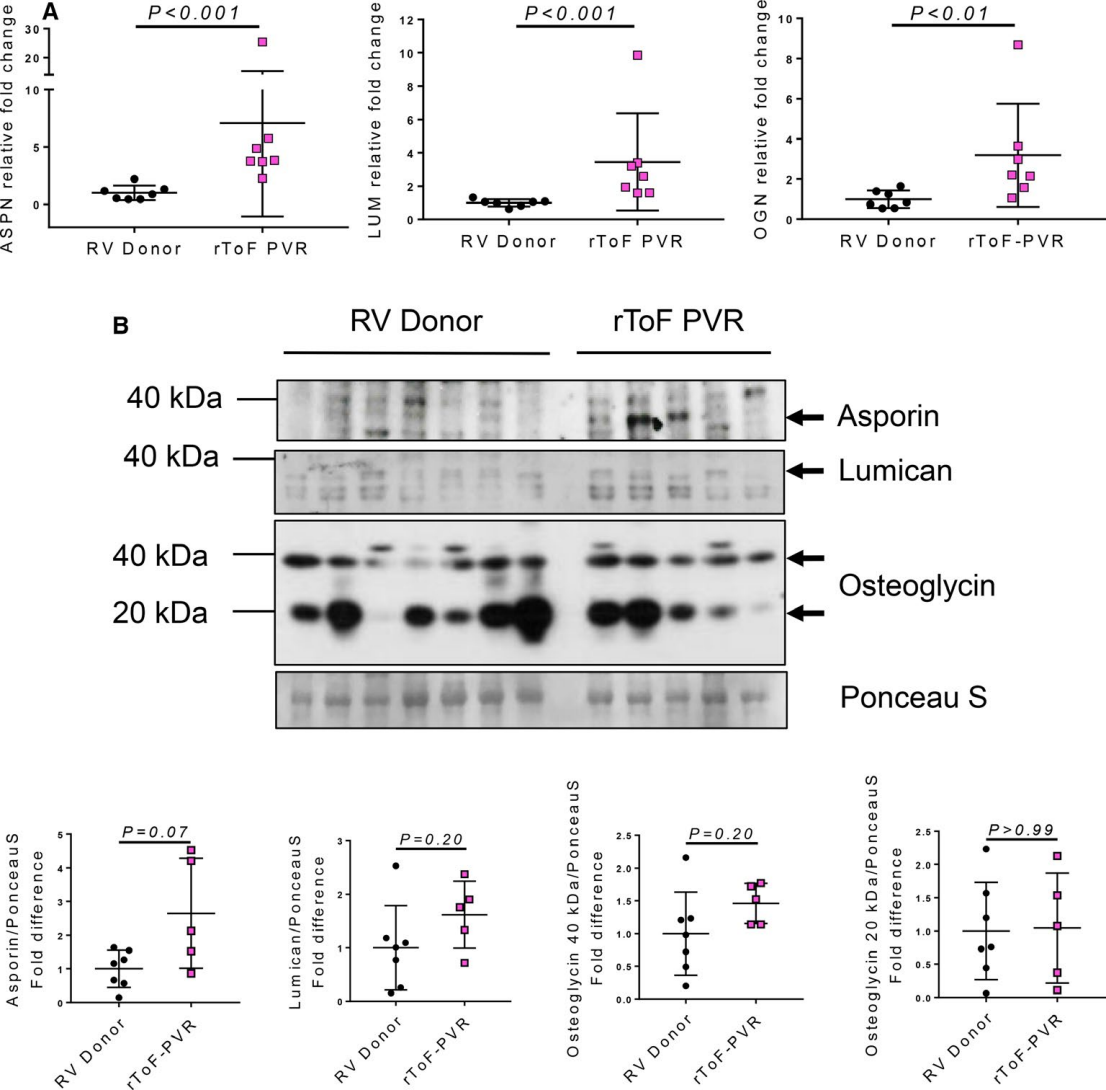
**Validation of small leucine‐rich proteoglycan (SLRP) gene and protein expression shows asporin to be upregulated in several myocardial samples from patients with repair of tetralogy of Fallot undergoing pulmonary valve replacement (rToF‐PVR).** **A**, The mRNA validation of gene expression was performed by quantitative polymerase chain reaction analysis for the genes encoding asporin (*ASPN*), lumican (*LUM*), and osteoglycin (*OGN*); n=7 per group. Each data point shows the result from 1 right ventricle (RV) donor or rToF‐PVR patient. Values are expressed as mean±SD, indicated *P* values were returned after performing the Mann–Whitney *U* test. The *P* values were unchanged if the single rToF‐PVR outlier was removed and the data were analyzed using the Student unpaired *t* test. **B**, Protein expression analysis for these genes was performed by western blotting using antibodies probing for asporin, lumican, and osteoglycin. Ponceau S shows equal loading of the gel lanes. Densitometry was performed for quantitation of protein abundance relative to ponceau S; RV donor, n=7; rToF‐PVR, n=5.

To check the transcriptomics data relating to SLRP expression, we used quantitative PCR, which confirmed that genes for asporin (*ASPN*), lumican (*LUM*) and osteoglycin (*OGN*) all underwent significant increases in mRNA expression in rToF‐PVR myocardium (Figure 7A). We then investigated their expression at the protein level. Western blotting showed a band corresponding to the predicted 43‐kDa molecular weight for asporin in rToF‐PVR samples and showed a trend toward increased expression (*P*=0.07) compared with RV donor samples. Lumican expression was similar between the groups, whereas osteoglycin, which is expressed in preprocessed (≈40 kDa) and postprocessed (≈20 kDa) forms,^24^ was expressed heterogeneously within and between groups (Figure 7B). Overall, changes in protein abundance of these SLRPs was absent, with the exception perhaps of asporin, which showed a tendency toward an increase in rToF‐PVR samples compared with RV donors, though this will need further examination.

**Figure 8:**
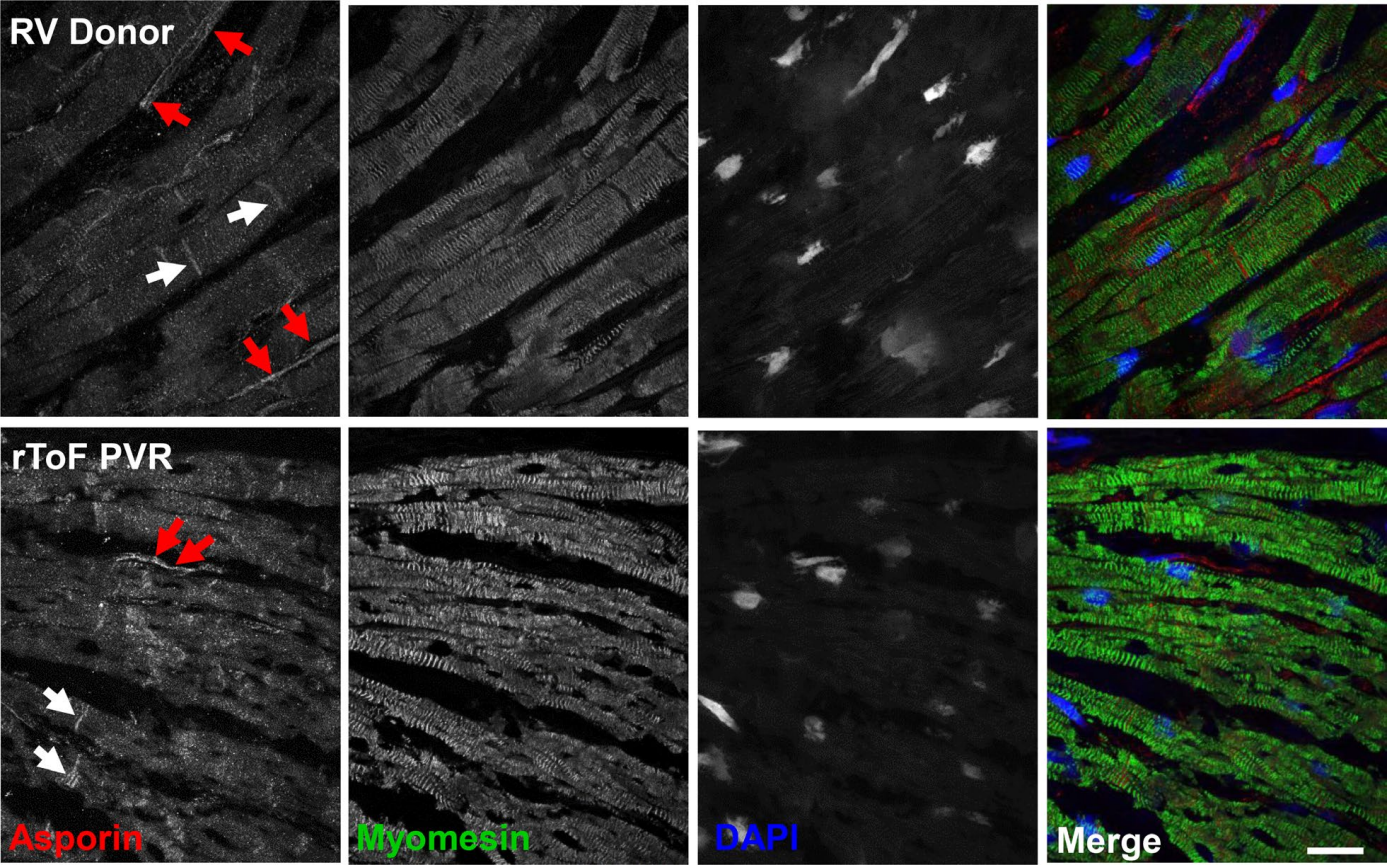
**Immunostaining of sections for asporin shows localization in extracellular domains and the intercalated disc in human right ventricle.** Asporin expression in right ventricle myocardium of both cohorts appeared in the extracellular domains in between the lateral membranes of cardiomyocytes suggestive of extraxcellular matrix localization (red arrows) or as regularly spaced bands indicative of intercalated disc staining (white arrows). Antibody to the sarcomere protein, myomesin, was used as a cardiomyocyte marker, and DAPI was used to stain nuclear DNA. Scale bar=10 μm. PVR indicates pulmonary valve replacement; rToF, repair of tetralogy of Fallot; RV, right ventricle

Immunofluorescence costaining for asporin and the myofilament protein marker myomesin showed that asporin was mainly localized to nonmyocyte populations but also appeared in the intercalated disc of the myocytes (Figure 8). Subjectively, no obvious differences between cohorts were observed.

## Discussion

This investigation is the first, to our knowledge, of myofilament function, tissue structure, and mRNA expression in the RV myocardium of adult, or near‐adult, patients with rToF requiring PVR. We found no differences in the passive stiffnesses or the active contractile properties of the myofilaments in rToF‐PVR myocytes compared with those in RV myocytes from donor hearts. We discovered subtle molecular differences in the ECM that may indicate an early phase of tissue remodeling in the RV of patients with rToF undergoing PVR; however, the myocardium of patients with rToF undergoing PVR, based on current clinical criteria, appears not to have undergone substantial remodeling at the cellular level, which should be beneficial for successful postoperative recovery.

### Functional Assessment of rToF‐PVR Cardiomyocytes

Previous studies have established that chronic dilation of the LV, such as that triggered by myocardial infarction or aortic regurgitation, leads to major changes to the contractile apparatus within the myocytes. For example, the passive stiffness of LV myocytes is increased because of changes in the expression and phosphorylation of the structural protein titin (which links actin and myosin filaments in the myofibril).^10^, ^12^ This would tend to increase the diastolic stiffness of the ventricle. The Ca^2+^ sensitivity of the myofibrils is increased because of reduced phosphorylation of the regulatory protein troponin I,^10^, ^25^ and this would tend to increase systolic contraction of the ventricle while slowing diastolic relaxation. The RV can also show disease‐dependent alterations within the myocytes that can influence the contractile state. Permeabilized myocytes from patients with pulmonary arterial hypertension and RV heart failure exhibit increased passive stiffness and maximum Ca^2+^‐activated force production compared with donor myocytes, and these changes could contribute to RV diastolic dysfunction in pulmonary arterial hypertension.^26^, ^27^ To our knowledge, there have been no similar studies on the functional properties of myocytes from the volume‐overloaded RV tissue of patients with rToF, taken at the time of PVR surgery. We found no difference in passive stiffness of rToF‐PVR permeabilized cardiac myocytes compared with myocytes from nondiseased RV tissue (Figure 1). Similarly, the steady‐state properties (maximum force production, Ca^2+^ sensitivity) and dynamic properties (*k*_tr_) of the myocytes during Ca^2+^ activation were the same as those from the RV donor controls. Therefore, we did not find any of the pathologic changes in myofibrillar function that have been seen previously in volume‐overloaded LV or in pressure‐overloaded RV. This result suggests that the rToF‐PVR myocytes we studied were structurally sound and that any structural remodeling of the myocytes had not occurred or had not reached a pathologic state detectable by our experiments. In summary, these myocytes were essentially "normal" and did not display a functional phenotype that has been associated with a severely diseased ventricle. Supporting this conclusion, although the RV was severely dilated in these patients, the RV ejection fraction was within the normal range.

### Subtle Changes in the ECM of the Remodeled Myocardium in Patients With rToF Undergoing PVR

ECM remodeling in which collagen content is increased is a cardinal feature of heart failure, as studied in LV.^28^ We observed that collagen content of myocardium was similar between rToF‐PVR and RV donor myocardium (Figure 2), which contradicts previous studies that showed apparent fibrosis by CMR imaging.^29^ This finding may reflect the differences in the selectivity of the 2 modes of imaging, as magnetic resonance imaging techniques rely on contrast agents that are thought to target scarred, inflamed, or remodeled areas, whereas picrosirius red staining very specifically detects collagen fibers. We discovered subtle changes in ECM gene expression in rToF‐PVR tissue compared with RV donor tissue. Tissue microarray revealed differences in gene expression between donor RV myocardium and rToF PVR myocardium in 601 transcripts when subjected to thresholds of *P*<0.04 and q=0.47. The selection of these thresholds was determined because samples became functionally grouped at this point. The gene list that was generated (Figure 4) revealed that "connective tissue diseases" were well represented, implicating the ECM changes in diseased tissue. Moreover, 3 of the top 10 transcripts from genes known to encode proteins belonged to the SLRP family. GO analysis of our transcriptomics data showed that immune‐related processes were also strongly implicated, and we hypothesized about whether inflammation contributed to an ECM disease milieu. However, analysis of pathway maps confirmed that the overwhelming majority of the players in the immune pathways were downregulated (Figure 5), making this unlikely. A note of caution is required with regard to the interpretation of our GO analysis because the threshold of q=0.47 is notably low and may have led to substantial false hits. However, this was unavoidable because stricter thresholds would have resulted in a very limited list of genes for GO analysis, and our intention was to validate hits that supported our hypothesis with further experimentation.

Proteoglycans are ECM‐modifying proteins that regulate the manner in which the core components of the ECM (ie, collagens) interact with each other and with the surface of cells.^30^ They are highly negatively charged molecules that can facilitate hydration of the extracellular spaces to form gel‐like structures such as the glycocalyx on the outer surface of cell membranes, potentially behaving as an ECM lubricant.^31^ The rheologic properties of proteoglycans are important in determining the surface mechanical properties of tissue, and there is a growing realization that dysregulation of proteoglycans may contribute to pathologic remodeling of the myocardium. For example, decorin and biglycan become highly expressed in models of hypertrophy and heart failure and are responsible for facilitating fibrillogenesis and fibrosis.^32^

We identified increased mRNA for the SLRP members asporin, lumican, and osteoglycin in rToF‐PVR myocardium. Of these, only asporin displayed a tendency for increased abundance at the protein level in rToF‐PVR samples. However, the functional significance of this is unclear at present because little is understood about the role of asporin in the heart. Upregulation of asporin has been observed during ECM remodeling in a pig model of myocardial infarction,^33^ but further work is required to establish the importance of asporin in RV function. In conclusion, our study provides evidence that the myocardium of patients with rToF undergoing PVR shows very little disturbance of myofilament function, histologic structure, and protein abundance, whereas gene‐expression changes may be indicative of an early response to volume overloading in these patients; however, this requires further investigation.

### Clinical Perspectives

Current indications for PVR are based on results of nonnvasive assessment, including volumetric and cardiac systolic function assessment with CMR imaging, clinical evaluation of symptoms, and objective assessment of exercise capacity using a cardiopulmonary exercising testing modality. However, it is difficult to predict which patients will most improve after PVR. In a previous study, we found that younger patients (aged <17.5 years) undergoing PVR exhibited greater 1‐year improvement of LV dynamics and exercise capacity than older patients. We suggested that the better outcome after PVR in younger patients could be due to a more compliant, less “diseased” RV, perhaps reflecting better myocardial protection at the time of primary repair and shorter exposure to volume overload.^7^ However, the present results, which were performed mostly on patients in the older age range, did not show evidence of substantial myocardial disease, at least at the cellular and ECM levels. Consequently, in these patients, ≈20 years after rToF and with severe pulmonary regurgitation, the contractile properties of the RV myofibrils and the ECM composition and structure were comparable to those in normal RV tissue. This suggests that the poorer outcome that we observed previously in the older patient group is unlikely to be caused by poorer reversibility of myocyte and ECM function. Current clinically based predictive indexes used to inform timing of PVR may be fit for purpose and are unlikely to subject patients to the risk of irreversible adverse remodeling of the RV.

We did find subtle changes in the expression of ECM genes in thepatients with rToF undergoing PVR. Whether these changes might signal the impending onset of detrimental changes in collagen structure and myofibril function remains a topic for future studies.

### Limitations

A main limitation of this study is the small number of available patients and the small amount of tissue that could be obtained from each, which did not allow us to test for any correlation between myocyte or ECM properties and age at the time of PVR. A more extensive study will be needed to address this potential correlation. Another potential limitation comes from the source of the tissue samples in the RV. Of necessity, we could obtain tissue only from the RV infundibulum when removing excessive residual muscle bundles. Although there is the potential that these samples might not be representative of the whole RV myocardium, these tissue sites have been exposed to the same chronic volume overload as the other areas within the RV, so the properties of their myocytes and ECM might be expected to be representative of those throughout the whole RV. Finally, the rToF‐PVR samples and the donor samples came from patients with different clinical histories and from 2 different countries (patients undergoing PVR in the United Kingdom; brain‐dead patients maintained on life support in Australia). However, using the same donor samples as a reference tissue, many studies have discovered substantial changes in myofilament function in other types of RV or LV disease,^21^ so we have confidence that our finding of no change in myofilament function between the donor and rToF‐PVR samples is a valid result.

## Sources of Funding

This work was supported by British Heart Foundation project grant PG/11/9/28705 (to Kentish, Frigiola, Tsang).

## Disclosures

None.

## Supporting information


**Table S1**
