# Supplementary material for: Right Ventricle Has Normal Myofilament Function But Shows Perturbations in the Expression of Extracellular Matrix Genes in Patients With Tetralogy of Fallot Undergoing Pulmonary Valve Replacement
Source: J Am Heart Assoc. 2020 Aug 1;9(16):e015342. doi: 10.1161/JAHA.119.015342 (PMC7660801; doi:10.1161/JAHA.119.015342)
Supplement: Supplementary file 1 — Table S1 [file JAH3-9-e015342-s001.pdf]

Supplemental Material

Table S1. Full List of genes retrieved by applying filtering thresholds of *P*=0.004 and q=0.47 to the rToF-PVR vs RV donor microarray.

Gene Symbol	Gene Title	p-value	q-value	Difference	Fold change
ASPN	asporin	0.00095587	0.301	2.222	4.666
GUCA1C	guanylate cyclase activator 1C	0.0007982	0.289	1.800	3.482
FRZB	frizzled-related protein	0.00019086	0.179	1.383	2.609
LUM	lumican	0.00043027	0.223	1.109	2.156
NPR3	natriuretic peptide receptor C/guanylate cyclase C (atrionatriuretic peptide receptor C)	0.00141221	0.339	1.027	2.038
OGN	osteoglycin	0.00252463	0.400	0.993	1.991
CRYM	crystallin, mu	0.00031254	0.207	0.944	1.924
C8orf4	chromosome 8 open reading frame 4	0.00229581	0.384	0.939	1.918
FCER1A	Fc fragment of IgE, high affinity I, receptor for; alpha polypeptide	0.00164494	0.351	0.900	1.866
FNDC1	fibronectin type III domain containing 1	2.28E-05	0.100	0.882	1.842
KCNJ2-AS1	KCNJ2 antisense RNA 1 (non-protein coding)	0.0004749	0.230	0.859	1.813
EDNRA	endothelin receptor type A	0.00394409	0.468	0.846	1.797
ALS2CR12	amyotrophic lateral sclerosis 2 (juvenile) chromosome region, candidate 12	0.00064006	0.256	0.842	1.792
PDE7B	phosphodiesterase 7B	0.00011862	0.149	0.840	1.790
EPHA7	EPH receptor A7	0.00335361	0.437	0.816	1.761
PLK2	polo-like kinase 2	0.0023682	0.388	0.790	1.729
MN1	meningioma (disrupted in balanced translocation) 1	0.00010048	0.140	0.789	1.728
LOC646168	uncharacterized LOC646168	0.00081887	0.292	0.763	1.697
SOX7	SRY (sex determining region Y)-box 7	0.00325966	0.436	0.713	1.639
NRARP	NOTCH-regulated ankyrin repeat protein	0.00061166	0.251	0.707	1.633
AGTR2	angiotensin II receptor, type 2	0.00216553	0.377	0.702	1.627
ECM2	extracellular matrix protein 2, female organ and adipocyte specific	0.00283864	0.421	0.685	1.608
IFIT2	interferon-induced protein with tetratricopeptide repeats 2	0.0006595	0.259	0.676	1.598
NREP	neuronal regeneration related protein homolog (rat)	0.00253667	0.400	0.661	1.581
LOC100506769	uncharacterized LOC100506769	0.00331014	0.437	0.648	1.567
LOC100270804	uncharacterized LOC100270804	0.00013112	0.156	0.631	1.549
HEG1	HEG homolog 1 (zebrafish)	6.17E-06	0.062	0.615	1.532
CCDC102B	coiled-coil domain containing 102B	0.00134726	0.338	0.586	1.501
TP53INP1	tumor protein p53 inducible nuclear protein 1	0.0033995	0.441	0.574	1.488
C10orf71	chromosome 10 open reading frame 71	0.00359906	0.452	0.557	1.472
PDE5A	phosphodiesterase 5A, cGMP-specific	0.00064754	0.258	0.553	1.468

CNTNAP3	contactin associated protein-like 3	0.00146161	0.340	0.534	1.448
OR10AD1	olfactory receptor, family 10, subfamily AD, member 1	0.00243262	0.394	0.534	1.448
SNORD116-26	small nucleolar RNA, C/D box 116-26	0.00372865	0.460	0.526	1.440
CPE	carboxypeptidase E	0.00021934	0.184	0.520	1.433
SMOC2	SPARC related modular calcium binding 2	0.00019092	0.179	0.513	1.427
HES1	hairy and enhancer of split 1, (Drosophila)	0.00277526	0.419	0.512	1.426
SOX4	SRY (sex determining region Y)-box 4	0.00318828	0.432	0.507	1.421
RN5S320	RNA, 5S ribosomal 320	0.00388418	0.464	0.497	1.411
SERTAD4	SERTA domain containing 4	0.00254978	0.401	0.496	1.410
MIR126	microRNA 126	0.00113114	0.313	0.495	1.409
KCNN3	potassium intermediate/small conductance calcium-activated channel, subfamily N, member 3	3.25E-05	0.115	0.489	1.404
HLTF	helicase-like transcription factor	0.00051927	0.238	0.486	1.401
TTC3P1	tetratricopeptide repeat domain 3 pseudogene 1	0.00231633	0.384	0.478	1.393
TCEAL7	transcription elongation factor A (SII)-like 7	0.00113883	0.313	0.451	1.367
PKD1L1	polycystic kidney disease 1 like 1	0.0003379	0.209	0.450	1.366
PIN4P1	protein (peptidylprolyl cis/trans isomerase) NIMA-interacting, 4 pseudogene 1	0.00035964	0.213	0.447	1.363
HIST1H2AG	histone cluster 1, H2ag /// histone cluster 1, H2ah /// histone cluster 1, H2ai /// histone cluster 1, H2ak /// histone cluster 1, H2al /// histone cluster 1, H2am	0.00090076	0.301	0.439	1.355
ZNF658B	zinc finger protein 658B, pseudogene	0.00111017	0.310	0.430	1.347
CCND1	cyclin D1	0.00051475	0.238	0.417	1.336
FAM129A	family with sequence similarity 129, member A	0.00266643	0.411	0.416	1.334
TET1	tet methylcytosine dioxygenase 1	0.00381215	0.461	0.406	1.325
FAM173B	family with sequence similarity 173, member B	0.00100551	0.303	0.393	1.313
LOC100506123	uncharacterized LOC100506123	0.00133229	0.337	0.390	1.310
ZDHHC15	zinc finger, DHHC-type containing 15	0.00034365	0.209	0.388	1.309
GUSBP2	glucuronidase, beta pseudogene 2 /// glucuronidase, beta pseudogene	0.00393989	0.468	0.386	1.306
ITIH5	inter-alpha-trypsin inhibitor heavy chain family, member 5	2.04E-05	0.096	0.385	1.306
CYSLTR2	cysteinyl leukotriene receptor 2	0.00027762	0.202	0.379	1.301
PTPN21	protein tyrosine phosphatase, non-receptor type 21	0.00097211	0.301	0.369	1.292
LOC100506123	uncharacterized LOC100506123	0.00164761	0.351	0.363	1.286
LRP2BP	LRP2 binding protein	0.00213774	0.375	0.362	1.286
LOC653501	zinc finger protein 658 pseudogene /// zinc finger protein 658 /// zinc finger protein 658B, pseudogene	0.00133441	0.337	0.360	1.283
LOC100653336	uncharacterized LOC100653336	0.00053163	0.242	0.354	1.278
PTCD2	pentatricopeptide repeat domain 2	0.0036253	0.453	0.352	1.276
LOC644135	uncharacterized LOC644135	0.00325367	0.436	0.345	1.270

DDIT3	DNA-damage-inducible transcript 3	0.00111135	0.310	0.341	1.267
CCND2	cyclin D2	0.00160437	0.348	0.332	1.259
ATP6AP1L	ATPase, H+ transporting, lysosomal accessory protein 1-like /// uncharacterized LOC645079	0.00209419	0.370	0.324	1.251
RGS4	regulator of G-protein signaling 4	0.00054283	0.244	0.323	1.251
SYTL2	synaptotagmin-like 2	0.00151955	0.344	0.317	1.246
TRAK1	trafficking protein, kinesin binding 1	0.00092904	0.301	0.313	1.242
C1QTNF7	C1q and tumor necrosis factor related protein 7	0.00227007	0.382	0.306	1.237
ARHGEF9	Cdc42 guanine nucleotide exchange factor (GEF) 9	0.00222169	0.379	0.292	1.224
PAFAH2	platelet-activating factor acetylhydrolase 2, 40kDa	0.00188145	0.367	0.288	1.221
SPTLC3	serine palmitoyltransferase, long chain base subunit 3	0.00247143	0.398	0.283	1.216
STAMBPL1	STAM binding protein-like 1	0.00129268	0.335	0.283	1.216
ZNF624	zinc finger protein 624	0.00206921	0.370	0.280	1.214
PROM1	prominin 1	6.62E-05	0.140	0.273	1.208
DPF3	D4, zinc and double PHD fingers, family 3	0.00247367	0.398	0.271	1.207
LOC100507388	uncharacterized LOC100507388	0.00138663	0.339	0.258	1.196
FAM210A	family with sequence similarity 210, member A	0.00286501	0.421	0.258	1.196
BPHL	biphenyl hydrolase-like (serine hydrolase)	0.00202258	0.370	0.252	1.191
ACOT2	acyl-CoA thioesterase 2	0.00257362	0.402	0.251	1.190
LACE1	lactation elevated 1	0.00149437	0.340	0.249	1.188
PHKA1	phosphorylase kinase, alpha 1 (muscle)	0.00280678	0.420	0.247	1.187
LOC100272216	uncharacterized LOC100272216	0.00297075	0.425	0.247	1.187
CCDC113	coiled-coil domain containing 113	0.00351859	0.446	0.244	1.184
FGD5	FYVE, RhoGEF and PH domain containing 5	0.00351253	0.446	0.241	1.182
PPARGC1A	peroxisome proliferator-activated receptor gamma, coactivator 1 alpha	0.00233201	0.384	0.240	1.181
COPS7B	COP9 constitutive photomorphogenic homolog subunit 7B (Arabidopsis)	0.00062408	0.253	0.240	1.181
ZNF222	zinc finger protein 222	0.00315506	0.432	0.234	1.176
ZNF724P	zinc finger protein 724, pseudogene /// zinc finger protein 724, pseudogene	0.00291976	0.425	0.234	1.176
SNORA38	small nucleolar RNA, H/ACA box 38	0.00148064	0.340	0.225	1.169
MDH1B	malate dehydrogenase 1B, NAD (soluble)	0.00139942	0.339	0.224	1.168
HAPLN1	hyaluronan and proteoglycan link protein 1	0.00270778	0.415	0.224	1.168
LNP1	leukemia NUP98 fusion partner 1	0.00366527	0.455	0.223	1.167
PCSK5	proprotein convertase subtilisin/kexin type 5	0.00120083	0.322	0.219	1.164
ZNF69	zinc finger protein 69	0.00328094	0.437	0.215	1.161
FOXP1	forkhead box P1	0.00251338	0.400	0.208	1.155
RANBP17	RAN binding protein 17	0.00198397	0.370	0.207	1.155

HCG27	HLA complex group 27 (non-protein coding)	0.00273573	0.416	0.201	1.149
MIR1302-8	microRNA 1302-8	0.00296469	0.425	0.195	1.145
PIK3C2B	phosphoinositide-3-kinase, class 2, beta polypeptide	0.00188992	0.367	0.184	1.136
CLEC14A	C-type lectin domain family 14, member A	0.00262711	0.407	0.181	1.133
PCP4	Purkinje cell protein 4	0.00295339	0.425	0.178	1.131
RNASEH2B-AS1	RNASEH2B antisense RNA 1 (non-protein coding)	0.00318168	0.432	0.169	1.124
PHLPP1	PH domain and leucine rich repeat protein phosphatase 1	0.00317698	0.432	0.165	1.121
KIAA1671	KIAA1671	0.00303305	0.426	0.158	1.116
FSIP2	fibrous sheath interacting protein 2	0.00336112	0.437	0.153	1.112
PPIL6	peptidylprolyl isomerase (cyclophilin)-like 6	0.00159178	0.347	0.148	1.108
ZNF440	zinc finger protein 440	0.00382757	0.461	0.142	1.103
MIR4766	microRNA 4766	0.00140201	0.339	0.140	1.102
AGAP2	ArfGAP with GTPase domain, ankyrin repeat and PH domain 2	0.00402441	0.470	0.140	1.102
TNF	tumor necrosis factor	0.00263523	0.408	0.128	1.092
AFF3	AF4/FMR2 family, member 3	0.00225583	0.381	0.127	1.092
OR51Q1	olfactory receptor, family 51, subfamily Q, member 1	0.00317672	0.432	0.124	1.090
FAM66C	family with sequence similarity 66, member C	0.00341159	0.441	0.124	1.090
TAS2R5	taste receptor, type 2, member 5	0.00188549	0.367	0.092	1.066
CCDC94	coiled-coil domain containing 94	0.00330715	0.437	-0.130	0.914
LAIR1	leukocyte-associated immunoglobulin-like receptor 1	0.00393227	0.468	-0.138	0.909
RBM47	RNA binding motif protein 47	0.00265163	0.409	-0.138	0.909
IL18BP	interleukin 18 binding protein	0.00175872	0.355	-0.143	0.906
DOCK2	dedicator of cytokinesis 2	0.00173664	0.355	-0.149	0.902
CMTM7	CKLF-like MARVEL transmembrane domain containing 7	0.0020104	0.370	-0.182	0.882
PCDH1	protocadherin 1	0.00351587	0.446	-0.184	0.880
LOC100288336	Putative uncharacterized protein ENSP00000383251	0.00090383	0.301	-0.186	0.879
EIF2B5-IT1	EIF2B5 intronic transcript 1 (non-protein coding)	0.00231478	0.384	-0.186	0.879
LPIN1	lipin 1	0.00168406	0.352	-0.193	0.875
C16orf57	chromosome 16 open reading frame 57	0.00199218	0.370	-0.199	0.871
PREX1	phosphatidylinositol-3,4,5-trisphosphate-dependent Rac exchange factor 1	0.00148326	0.340	-0.204	0.868
SELPLG	selectin P ligand	0.00134962	0.338	-0.206	0.867
NPL	N-acetylneuraminate pyruvate lyase (dihydrodipicolinate synthase)	0.00013234	0.156	-0.219	0.859
CHL1-AS2	CHL1 antisense RNA 2 (non-protein coding)	0.00191653	0.368	-0.230	0.853
GRINA	glutamate receptor, ionotropic, N-methyl D-aspartate-associated protein 1 (glutamate binding)	0.00252756	0.400	-0.234	0.851
TMEM236	transmembrane protein 236	0.00091526	0.301	-0.236	0.849

SLC4A7	solute carrier family 4, sodium bicarbonate cotransporter, member 7	0.00306824	0.427	-0.237	0.849
TMEM236	transmembrane protein 236	0.00032542	0.207	-0.239	0.848
JMJD6	jumonji domain containing 6	0.00104748	0.308	-0.240	0.847
NT5DC2	5'-nucleotidase domain containing 2	0.00139839	0.339	-0.243	0.845
EMILIN2	elastin microfibril interfacer 2	0.00307184	0.427	-0.246	0.843
MT1IP	metallothionein 1I, pseudogene	0.0020501	0.370	-0.248	0.842
WNK3	WNK lysine deficient protein kinase 3	0.00283373	0.421	-0.249	0.841
SERPINA5	serpin peptidase inhibitor, clade A (alpha-1 antiproteinase, antitrypsin), member 5	0.00093116	0.301	-0.249	0.841
NCF4	neutrophil cytosolic factor 4, 40kDa	0.0013664	0.338	-0.252	0.840
TMBIM1	transmembrane BAX inhibitor motif containing 1	0.00105118	0.308	-0.254	0.838
TRHDE	thyrotropin-releasing hormone degrading enzyme	0.00207115	0.370	-0.255	0.838
SLA	Src-like-adaptor	0.00019947	0.179	-0.267	0.831
PRKCD	protein kinase C, delta	0.00306934	0.427	-0.267	0.831
NAGA	N-acetylgalactosaminidase, alpha-	0.00019532	0.179	-0.268	0.831
ALOX5	arachidonate 5-lipoxygenase	0.00325725	0.436	-0.268	0.831
ATG9A	autophagy related 9A	0.00175925	0.355	-0.270	0.829
TMEM204	transmembrane protein 204	0.00115444	0.314	-0.271	0.829
CSTA	cystatin A (stefin A)	2.81E-05	0.104	-0.273	0.828
C1QA	complement component 1, q subcomponent, A chain	0.00345607	0.443	-0.275	0.827
GGT5	gamma-glutamyltransferase 5	0.00147036	0.340	-0.275	0.826
HCK	hemopoietic cell kinase	0.0009889	0.302	-0.276	0.826
WNK1	WNK lysine deficient protein kinase 1	0.00353478	0.447	-0.277	0.825
TMBIM6	transmembrane BAX inhibitor motif containing 6	0.00037047	0.213	-0.280	0.823
SOAT1	sterol O-acyltransferase 1	0.0034374	0.443	-0.285	0.821
NUDT16	nudix (nucleoside diphosphate linked moiety X)-type motif 16	0.00242874	0.394	-0.289	0.818
ADIPOR2	adiponectin receptor 2	0.00400447	0.470	-0.291	0.817
NPC1	Niemann-Pick disease, type C1	0.00173163	0.355	-0.299	0.813
MAP3K6	mitogen-activated protein kinase kinase kinase 6	0.00137857	0.339	-0.300	0.812
STAB1	stabilin 1	0.00159276	0.347	-0.302	0.811
SPTSSA	serine palmitoyltransferase, small subunit A	0.00083432	0.293	-0.314	0.805
H2AFZ	H2A histone family, member Z	0.0005156	0.238	-0.314	0.804
FCN3	ficolin (collagen/fibrinogen domain containing) 3 (Hakata antigen)	0.00225546	0.381	-0.317	0.803
NEDD9	neural precursor cell expressed, developmentally down-regulated 9	0.00097144	0.301	-0.322	0.800
JAK1	Janus kinase 1	0.00335459	0.437	-0.327	0.797
ZNF366	zinc finger protein 366	0.00165299	0.351	-0.327	0.797
SLC1A5	solute carrier family 1 (neutral amino acid transporter), member 5	0.00084604	0.294	-0.335	0.793

CTSB	cathepsin B	0.00180406	0.360	-0.335	0.793
MTCH1	mitochondrial carrier 1	0.0032598	0.436	-0.341	0.790
EFNB1	ephrin-B1	0.0020812	0.370	-0.348	0.786
TOB2	transducer of ERBB2, 2	0.00165455	0.351	-0.355	0.782
IL15RA	interleukin 15 receptor, alpha	0.00054114	0.244	-0.358	0.780
CR1	complement component (3b/4b) receptor 1 (Knops blood group)	0.00020094	0.179	-0.359	0.780
LOC100129027	uncharacterized LOC100129027	0.0004474	0.230	-0.360	0.779
IP6K3	inositol hexakisphosphate kinase 3	0.00090269	0.301	-0.365	0.776
C5orf62	chromosome 5 open reading frame 62	0.00355075	0.447	-0.371	0.773
KLF9	Kruppel-like factor 9	0.00374162	0.460	-0.372	0.773
C1QTNF1	C1q and tumor necrosis factor related protein 1	0.0017507	0.355	-0.373	0.772
EDN1	endothelin 1	0.00293656	0.425	-0.374	0.772
FCGR2B	Fc fragment of IgG, low affinity IIb, receptor (CD32)	0.0018078	0.360	-0.380	0.768
C10orf10	chromosome 10 open reading frame 10	0.00256911	0.402	-0.382	0.768
DDIT4	DNA-damage-inducible transcript 4	0.00060651	0.251	-0.382	0.767
C1RL	complement component 1, r subcomponent-like	0.00221786	0.379	-0.383	0.767
IL18R1	interleukin 18 receptor 1	0.00026271	0.194	-0.392	0.762
CD180	CD180 molecule	0.00208047	0.370	-0.395	0.760
ADORA3	adenosine A3 receptor	0.00286569	0.421	-0.403	0.757
PHF17	PHD finger protein 17	7.48E-05	0.140	-0.404	0.756
LGMN	legumain	0.00201783	0.370	-0.410	0.753
MAP3K8	mitogen-activated protein kinase kinase kinase 8	0.000301	0.207	-0.415	0.750
PPP3CC	protein phosphatase 3, catalytic subunit, gamma isozyme	0.00198953	0.370	-0.420	0.748
MPP3	membrane protein, palmitoylated 3 (MAGUK p55 subfamily member 3)	0.00097871	0.301	-0.423	0.746
TMTC1	transmembrane and tetratricopeptide repeat containing 1	0.0013494	0.338	-0.425	0.745
GPR4	G protein-coupled receptor 4	0.00404862	0.470	-0.428	0.743
IFNGR1	interferon gamma receptor 1	0.00012687	0.156	-0.429	0.743
SMAP2	small ArfGAP2	4.88E-05	0.137	-0.431	0.742
MT1B	metallothionein 1B	0.00278246	0.419	-0.433	0.741
VAMP8	vesicle-associated membrane protein 8 (endobrevin)	0.00111191	0.310	-0.436	0.739
IL17RA	interleukin 17 receptor A	0.0004218	0.223	-0.438	0.738
HCST	hematopoietic cell signal transducer	0.00032605	0.207	-0.443	0.736
IFITM4P	interferon induced transmembrane protein 4 pseudogene	0.00095487	0.301	-0.463	0.725
CALR	calreticulin	0.00276903	0.419	-0.465	0.724
IFITM4P	interferon induced transmembrane protein 4 pseudogene	0.00122516	0.325	-0.470	0.722
MT1DP	metallothionein 1D, pseudogene /// metallothionein 1D, pseudogene	0.00206797	0.370	-0.478	0.718

CEBPD	CCAAT/enhancer binding protein (C/EBP), delta	0.00214413	0.375	-0.479	0.717
STK17B	serine/threonine kinase 17b	0.00057997	0.247	-0.479	0.717
GNMT	glycine N-methyltransferase	0.0018093	0.360	-0.480	0.717
C1QB	complement component 1, q subcomponent, B chain	0.00159411	0.347	-0.492	0.711
TMEM45A	transmembrane protein 45A	0.00035573	0.213	-0.493	0.711
RNF144B	ring finger protein 144B	0.00285125	0.421	-0.500	0.707
IFITM3	interferon induced transmembrane protein 3	0.0039756	0.470	-0.509	0.703
S1PR3	sphingosine-1-phosphate receptor 3	0.0021775	0.377	-0.511	0.702
AIF1	allograft inflammatory factor 1	0.00087948	0.297	-0.514	0.700
FOXO3	forkhead box O3	0.00017806	0.179	-0.522	0.697
CPM	carboxypeptidase M	3.93E-05	0.124	-0.523	0.696
NFKBIA	nuclear factor of kappa light polypeptide gene enhancer in B-cells inhibitor, alpha	0.00084533	0.294	-0.525	0.695
NFKBIZ	nuclear factor of kappa light polypeptide gene enhancer in B-cells inhibitor, zeta	0.00251236	0.400	-0.538	0.689
IFITM4P	interferon induced transmembrane protein 4 pseudogene	0.0003967	0.220	-0.541	0.687
FOSL2	FOS-like antigen 2	0.0002609	0.194	-0.542	0.687
AASS	aminoadipate-semialdehyde synthase	0.00291745	0.425	-0.548	0.684
MTHFD2	methylenetetrahydrofolate dehydrogenase (NADP+ dependent) 2, methenyltetrahydrofolate cyclohydrolase	0.00096372	0.301	-0.552	0.682
LRP1	low density lipoprotein receptor-related protein 1	0.00184927	0.364	-0.560	0.679
CD209	CD209 molecule	0.00122891	0.325	-0.563	0.677
LDHA	lactate dehydrogenase A	0.00030731	0.207	-0.571	0.673
IRAK3	interleukin-1 receptor-associated kinase 3	0.00074417	0.279	-0.574	0.672
BCAT1	branched chain amino-acid transaminase 1, cytosolic	0.00087519	0.297	-0.576	0.671
IFITM2	interferon induced transmembrane protein 2	0.0020637	0.370	-0.579	0.669
TGFBR2	transforming growth factor, beta receptor II (70/80kDa)	0.00106045	0.308	-0.587	0.666
TNFRSF1A	tumor necrosis factor receptor superfamily, member 1A	0.00153604	0.346	-0.587	0.666
SAT1	spermidine/spermine N1-acetyltransferase 1	0.0021567	0.376	-0.589	0.665
CCL18	chemokine (C-C motif) ligand 18 (pulmonary and activation-regulated)	0.00197433	0.370	-0.592	0.663
SLCO4A1	solute carrier organic anion transporter family, member 4A1	0.00077152	0.286	-0.604	0.658
RGCC	regulator of cell cycle	0.00099195	0.302	-0.606	0.657
AREG	amphiregulin /// amphiregulin B	0.00382854	0.461	-0.608	0.656
LAMB1	laminin, beta 1	0.00167156	0.351	-0.608	0.656
PYGL	phosphorylase, glycogen, liver	0.00056939	0.246	-0.617	0.652
FCGR2A	Fc fragment of IgG, low affinity IIa, receptor (CD32)	0.00106489	0.308	-0.619	0.651
RHOU	ras homolog family member U	0.00061645	0.251	-0.622	0.650
ZBTB16	zinc finger and BTB domain containing 16	0.00182702	0.361	-0.624	0.649

IGFBP4	insulin-like growth factor binding protein 4	0.00223569	0.380	-0.625	0.649
SNAI2	snail homolog 2 (Drosophila)	0.00032442	0.207	-0.626	0.648
CPEB4	cytoplasmic polyadenylation element binding protein 4	8.89E-05	0.140	-0.633	0.645
DCLK1	doublecortin-like kinase 1	0.0033391	0.437	-0.633	0.645
MYC	v-myc myelocytomatosis viral oncogene homolog (avian)	0.00201717	0.370	-0.634	0.644
SLC9A9	solute carrier family 9, subfamily A (NHE9, cation proton antiporter 9), member 9	0.00121644	0.324	-0.644	0.640
SLC1A3	solute carrier family 1 (glial high affinity glutamate transporter), member 3	0.00023809	0.186	-0.645	0.640
PDGFRA	platelet-derived growth factor receptor, alpha polypeptide	0.0015716	0.347	-0.658	0.634
C1orf162	chromosome 1 open reading frame 162	0.00048959	0.230	-0.659	0.633
NCKAP1L	NCK-associated protein 1-like	0.00020059	0.179	-0.669	0.629
MERTK	c-mer proto-oncogene tyrosine kinase	0.00023205	0.186	-0.679	0.625
IL2RA	interleukin 2 receptor, alpha	0.00057691	0.247	-0.679	0.624
CLEC4E	C-type lectin domain family 4, member E	0.00042492	0.223	-0.680	0.624
STAT3	signal transducer and activator of transcription 3 (acute-phase response factor)	0.0005666	0.246	-0.683	0.623
MT1E	metallothionein-2-like /// metallothionein 1E	0.0004781	0.230	-0.684	0.622
LINC00341	long intergenic non-protein coding RNA 341	0.00029182	0.207	-0.687	0.621
SLC38A2	solute carrier family 38, member 2	0.00228489	0.383	-0.695	0.618
C5AR1	complement component 5a receptor 1	0.00045703	0.230	-0.700	0.616
FCGR2C	Fc fragment of IgG, low affinity IIc, receptor for (CD32) (gene/pseudogene)	0.0002092	0.179	-0.704	0.614
MT1H	metallothionein 1H	0.00101652	0.305	-0.712	0.611
HCLS1	hematopoietic cell-specific Lyn substrate 1	0.0004215	0.223	-0.714	0.610
CD53	CD53 molecule	0.00046111	0.230	-0.719	0.607
MAFB	v-maf musculoaponeurotic fibrosarcoma oncogene homolog B (avian)	5.81E-05	0.137	-0.728	0.604
TSPYL2	TSPY-like 2	0.000188	0.179	-0.731	0.602
GPR183	G protein-coupled receptor 183	0.00146787	0.340	-0.737	0.600
LCP1	lymphocyte cytosolic protein 1 (L-plastin)	0.000312	0.207	-0.737	0.600
GPX3	glutathione peroxidase 3 (plasma)	0.00078525	0.287	-0.745	0.597
TUBA3C	tubulin, alpha 3c /// tubulin, alpha 3d	2.47E-05	0.102	-0.753	0.593
MAN1A1	mannosidase, alpha, class 1A, member 1	0.00348865	0.445	-0.759	0.591
BCL6	B-cell CLL/lymphoma 6	0.00029387	0.207	-0.764	0.589
NAMPT	nicotinamide phosphoribosyltransferase	0.00383478	0.461	-0.781	0.582
FCGR1B	Fc fragment of IgG, high affinity Ib, receptor (CD64) /// Fc fragment of IgG, high affinity Ic, receptor (CD64), pseudogene	0.00204752	0.370	-0.792	0.577
MS4A6A	membrane-spanning 4-domains, subfamily A, member 6A	0.00205473	0.370	-0.798	0.575
GPR34	G protein-coupled receptor 34	0.00158982	0.347	-0.798	0.575

C1R	complement component 1, r subcomponent /// complement component 1, r subcomponent	0.00016103	0.177	-0.809	0.571
C3AR1	complement component 3a receptor 1	0.00071684	0.273	-0.836	0.560
MGST1	microsomal glutathione S-transferase 1	8.88E-06	0.062	-0.838	0.559
LPCAT3	lysophosphatidylcholine acyltransferase 3	0.00034145	0.209	-0.840	0.559
ZNF189	zinc finger protein 189	8.21E-06	0.062	-0.849	0.555
FCGR1A	Fc fragment of IgG, high affinity Ia, receptor (CD64) /// Fc fragment of IgG, high affinity Ib, receptor (CD64)	0.00236892	0.388	-0.871	0.547
SAMHD1	SAM domain and HD domain 1	0.00114078	0.313	-0.890	0.539
GADD45B	growth arrest and DNA-damage-inducible, beta	0.00102324	0.305	-0.891	0.539
ALOX5AP	arachidonate 5-lipoxygenase-activating protein	9.32E-05	0.140	-0.899	0.536
MT1G	metallothionein 1G	0.00320585	0.433	-0.902	0.535
TLR2	toll-like receptor 2	8.91E-05	0.140	-0.934	0.523
C1QC	complement component 1, q subcomponent, C chain	0.00129441	0.335	-0.939	0.522
CCR1	chemokine (C-C motif) receptor 1	6.21E-05	0.140	-0.948	0.518
EFEMP1	EGF containing fibulin-like extracellular matrix protein 1	0.00056958	0.246	-0.956	0.516
FCGR3A	Fc fragment of IgG, low affinity IIIa, receptor (CD16a)	0.00034213	0.209	-0.964	0.513
IL1RL1	interleukin 1 receptor-like 1	0.00081458	0.292	-0.979	0.507
ERRFI1	ERBB receptor feedback inhibitor 1	7.32E-05	0.140	-0.986	0.505
MT2A	metallothionein 2A	0.0001734	0.179	-1.002	0.499
NNMT	nicotinamide N-methyltransferase	0.00098908	0.302	-1.015	0.495
FST	follistatin	0.00020189	0.179	-1.022	0.492
PLTP	phospholipid transfer protein	0.0004028	0.220	-1.052	0.482
EDNRB	endothelin receptor type B	0.00025294	0.192	-1.077	0.474
ADAMTS9	ADAM metallopeptidase with thrombospondin type 1 motif, 9	0.00221748	0.379	-1.079	0.473
MT1JP	metallothionein 1J, pseudogene	0.00045811	0.230	-1.102	0.466
GLUL	glutamate-ammonia ligase	7.27E-05	0.140	-1.104	0.465
OSMR	oncostatin M receptor	0.00023416	0.186	-1.109	0.464
CDH19	cadherin 19, type 2	0.00290402	0.425	-1.111	0.463
CD68	CD68 molecule	0.000193	0.179	-1.117	0.461
PLIN2	uncharacterized LOC100509484 /// perilipin 2	5.72E-05	0.137	-1.127	0.458
LAPTM5	lysosomal protein transmembrane 5	0.00074984	0.280	-1.145	0.452
CYBB	cytochrome b-245, beta polypeptide	0.00035589	0.213	-1.160	0.448
S100A8	S100 calcium binding protein A8	0.00010972	0.141	-1.163	0.447
MT1CP	metallothionein 1C, pseudogene	0.00016561	0.179	-1.187	0.439
FPR1	formyl peptide receptor 1	0.00125227	0.327	-1.207	0.433

MT1X	metallothionein 1X	0.00016819	0.179	-1.214	0.431
TUBA3C	tubulin, alpha 3c /// tubulin, alpha 3d	3.79E-06	0.053	-1.217	0.430
MS4A4A	membrane-spanning 4-domains, subfamily A, member 4A	0.00253196	0.400	-1.225	0.428
S100A12	S100 calcium binding protein A12	0.00232406	0.384	-1.260	0.418
BTG2	BTG family, member 2	8.22E-05	0.140	-1.264	0.416
C3	complement component 3	0.00102626	0.305	-1.331	0.397
CTSC	cathepsin C	0.00194506	0.370	-1.339	0.395
SRPX	sushi-repeat containing protein, X-linked	4.75E-06	0.056	-1.358	0.390
FCER1G	Fc fragment of IgE, high affinity I, receptor for; gamma polypeptide	0.00047948	0.230	-1.362	0.389
ABRA	actin-binding Rho activating protein	0.0025267	0.400	-1.383	0.384
TUBA3E	tubulin, alpha 3e	6.61E-05	0.140	-1.394	0.381
MT1M	metallothionein 1M	4.04E-05	0.124	-1.470	0.361
RNASE2	ribonuclease, RNase A family, 2 (liver, eosinophil-derived neurotoxin)	0.00020752	0.179	-1.486	0.357
ZFP36	zinc finger protein 36, C3H type, homolog (mouse)	0.00048998	0.230	-1.530	0.346
MRC1	mannose receptor, C type 1	0.00020262	0.179	-1.605	0.329
RASD1	RAS, dexamethasone-induced 1	2.77E-05	0.104	-1.673	0.314
VSIG4	V-set and immunoglobulin domain containing 4	0.0001281	0.156	-1.738	0.300
FKBP5	FK506 binding protein 5	3.90E-05	0.124	-1.770	0.293
S100A9	S100 calcium binding protein A9	1.89E-05	0.095	-1.800	0.287
F13A1	coagulation factor XIII, A1 polypeptide	5.59E-05	0.137	-1.812	0.285
LYVE1	lymphatic vessel endothelial hyaluronan receptor 1	4.51E-05	0.132	-1.929	0.263
PLA2G2A	phospholipase A2, group IIA (platelets, synovial fluid)	1.84E-07	0.006	-2.035	0.244
SERPINA3	serpin peptidase inhibitor, clade A (alpha-1 antiproteinase, antitrypsin), member 3	9.75E-06	0.062	-2.184	0.220
CD163	CD163 molecule	2.43E-06	0.043	-2.374	0.193
MT1A	metallothionein 1A	1.52E-05	0.083	-2.882	0.136
